# On the design and fabrication of nanoliter-volume hanging drop networks

**DOI:** 10.1038/s41378-024-00788-0

**Published:** 2024-10-16

**Authors:** Matthew Wester, Jongwon Lim, Liliana Khaertdinova, Sriya Darsi, Neel Donthamsetti, Glennys Mensing, George Vasmatzis, Panos Anastasiadis, Enrique Valera, Rashid Bashir

**Affiliations:** 1 Department of Bioengineering, University of Illinois Urbana-Champaign, Urbana, IL 61801 USA; 2 Nick Holonyak Jr. Micro and Nanotechnology Laboratory, University of Illinois Urbana-Champaign, Urbana, IL 61801 USA; 3 Center for Individualized Medicine, Mayo Clinic, Rochester, MN 55905 USA; 4 Mayo-Illinois Alliance for Technology-Based Healthcare, Urbana, IL 61801 USA; 5 Department of Cancer Biology, Mayo Clinic, Jacksonville, FL 32224 USA; 6 Carl R. Woese Institute for Genomic Biology, University of Illinois at Urbana-Champaign, Urbana, IL 61801 USA; 7 Biomedical Research Center, Carle Foundation Hospital, Urbana, IL 61801 USA; 8 Cancer Center at Illinois, University of Illinois at Urbana-Champaign, Urbana, IL 61801 USA; 9 Department of Biomedical and Translation Science, Carle Illinois College of Medicine, University of Illinois at Urbana-Champaign, Urbana, IL 61801 USA; 10 Chan Zuckerberg Biohub Chicago, Chicago, IL 60642 USA; 11 Department of Electrical and Computer Engineering, University of Illinois at Urbana-Champaign, Urbana, IL 61801 USA; 12 Department of Mechanical Science and Engineering, University of Illinois at Urbana-Champaign, Urbana, IL 61801 USA; 13 Department of Materials Science and Engineering, University of Illinois at Urbana-Champaign, Urbana, IL 61801 USA

**Keywords:** Engineering, Materials science

## Abstract

Hanging drop cultures provide a favorable environment for the gentle, gel-free formation of highly uniform three-dimensional cell cultures often used in drug screening applications. Initial cell numbers can be limited, as with primary cells provided by minimally invasive biopsies. Therefore, it can be beneficial to divide cells into miniaturized arrays of hanging drops to supply a larger number of samples. Here, we present a framework for the miniaturization of hanging drop networks to nanoliter volumes. The principles of a single hanging drop are described and used to construct the fundamental equations for a microfluidic system composed of multiple connected drops. Constitutive equations for the hanging drop as a nonlinear capacitive element are derived for application in the electronic-hydraulic analogy, forming the basis for more complex, time-dependent numerical modeling of hanging drop networks. This is supplemented by traditional computational fluid dynamics simulation to provide further information about flow conditions within the wells. A fabrication protocol is presented and demonstrated for creating transparent, microscale arrays of pinned hanging drops. A custom interface, pressure-based fluidic system, and environmental chamber have been developed to support the device. Finally, fluid flow on the chip is demonstrated to align with expected behavior based on the principles derived for hanging drop networks. Challenges with the system and potential areas for improvement are discussed. This paper expands on the limited body of hanging drop network literature and provides a framework for designing, fabricating, and operating these systems at the microscale.

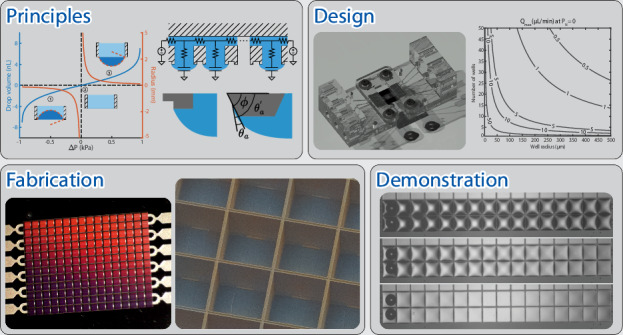

## Introduction

Hanging drop culture was pioneered by Robert Koch in the 1870s for the study of *Bacillus anthracis* in an enclosed environment amenable to imaging^1^. The hanging drop quickly became a recognized bacterial culture technique and remains in use, especially to observe bacterial motility^2,3^. The method was adapted to eukaryotic cell culture in 1907 to observe the development of nerve fibers from frog embryonic cells^4^. With the introduction of antibiotics to cell culture, simple protocols based on the inversion of a petri dish became feasible^5^. As with other three-dimensional culture techniques, spheroids formed in hanging drops have been shown to more closely replicate in vivo conditions when compared to traditional monolayer culture^6,7^. Hanging drop culture has been of particular interest because the gravity-driven cell assembly leads to highly uniform spheroids of adjustable size without the need for a gel or other supporting scaffold. Because of the resulting low shear forces, hanging drop platforms are well suited to culture with sensitive cell types, including certain primary cells^8^. They have also been utilized for coculture^9^.

Hanging drop platforms present some challenges. Because of the high surface area, they tend to evaporate more quickly than traditional plates or flasks, leading to increased media osmolality that necessitates frequent media exchange. Further complicating matters, the droplets are sensitive to environmental perturbations, especially during spheroid formation and media exchange. Because of these challenges with completing assays on the platform, spheroids are typically removed for imaging and endpoint analysis. And because of the need for careful manual steps, throughput is low. To improve on this, several hanging drop array platforms have been developed over the past two decades. These have introduced various features to improve droplet stability, mitigate evaporation issues, and execute specific assays.

Two commercial platforms have become available based on the common 96 and 384-well plate formats^10–12^. These include modified wells that allow cell solutions to be pipetted into openings on top of the chip. This provides a major improvement in addressability, with the possibility of integration with robotic fluid handling units. The commercial options also include a lower plate with fluid reservoirs to humidify the surrounding air and reduce evaporation. However, imaging and manual fluid exchange are still difficult, so spheroids are often formed on the platform and then transferred to traditional wells for further operations.

To remove the need for manual interaction with the wells, a few platforms have included channels for media exchange. Frey et al. demonstrated a reconfigurable network of millimeter-scale droplets in polydimethylsiloxane (PDMS)^13^. In this example, rows of four wells were connected in series by perfusion channels. Notably, the lower surface of the channels was not enclosed, resulting in a continuous exposed surface across the device. Multiple configurations were utilized to introduce cell solutions to the wells. After allowing for spheroid formation over ~60 h, flow driven by a syringe or peristaltic pump was initiated in the perfusion channels. This platform was applied to drug exposure and bioactivation coculture assays. A 384-well plate version without active perfusion was also demonstrated. In a similar platform, Yazdi et al. and Wu et al. implemented an integrated pump and a control system based on the aspiration of fluid through a needle valve, respectively^14,15^. Rousset et al. implemented a circuit-based model for analysis and studied the behavior of cells and beads in this type of open platform in more detail^16,17^. To facilitate media exchange in a fully open microfluidic platform, de Groot et al. designed a machined two-well system composed of a loading well and a culture well, with transfer driven by Laplace pressure^18^. This was primarily aimed at reducing the shear stress exposure in the culture well for application with easily disturbed suspension cells. Rodoplu et al. designed a combined polystyrene-PDMS hanging drop array device for forming spheroids of two distinct cell types and then exposing the spheroids in shared chambers^19^. Media was exchanged by simultaneously pipetting into one port and out of another in 50 µL increments.

While these papers have demonstrated various configurations of hanging drop networks, exploration of the dynamics of multiple drop networks under active perfusion has been limited. Critically, all previous connected arrays have utilized millimeter-scale droplets. As a result, they have not been able to take advantage of many of the key benefits of microfluidic cell culture, namely low reagent volumes, fast diffusion, and rapid spheroid formation. Only one previous paper (from our group) has demonstrated nanoliter-volume hanging drop arrays for cell culture^20^. Because of the lack of integrated microfluidics, however, media replacement and on-chip assays were challenging. In this paper, we present important design considerations based on system modeling, describe a novel fabrication process, and provide a basic demonstration of the functional characteristics of the first nanoliter-volume hanging drop network.

## Results and discussion

### Hanging drop principle

The geometry of the hanging drop is described by the Young–Laplace equation^21^:

1 $$\Delta P=\sigma \left(\frac{1}{{r}_{1}}+\frac{1}{{r}_{2}}\right)$$

where ΔP is the pressure difference across an interface with constant interfacial tension, σ, and principal radii of curvature, ${r}_{1}$ and ${r}_{2}$. This equation remains valid down to nanometer-scale droplets^22^. The Bond number can be used to assess the relative effect of surface tension and gravity in a microfluidic system. This is defined as^23^:

2 $$\text{Bo}=\frac{\Delta \rho g{L}^{2}}{\sigma }$$

where $\Delta \rho$ is the difference in density across the fluid-fluid interface, $g$ is gravitational acceleration, $L$ is the characteristic length, and $\sigma$ is the surface tension. Bond numbers less than one indicate that surface tension effects dominate. For an air-water hanging drop, a Bond number of one occurs at a characteristic length of approximately 8.6 mm. As the drops demonstrated here have a significantly smaller characteristic length (L ~150 µm, Bo ~0.0031), we will neglect any droplet deformation due to gravity. To provide an intuitive explanation of hanging drop systems, we will also consider the case of axially symmetric droplets, for which ${r}_{1}$ = ${r}_{2}$ and Eq. (1) can be reduced to $\Delta P=2\sigma /r$.

**Fig. 1:**
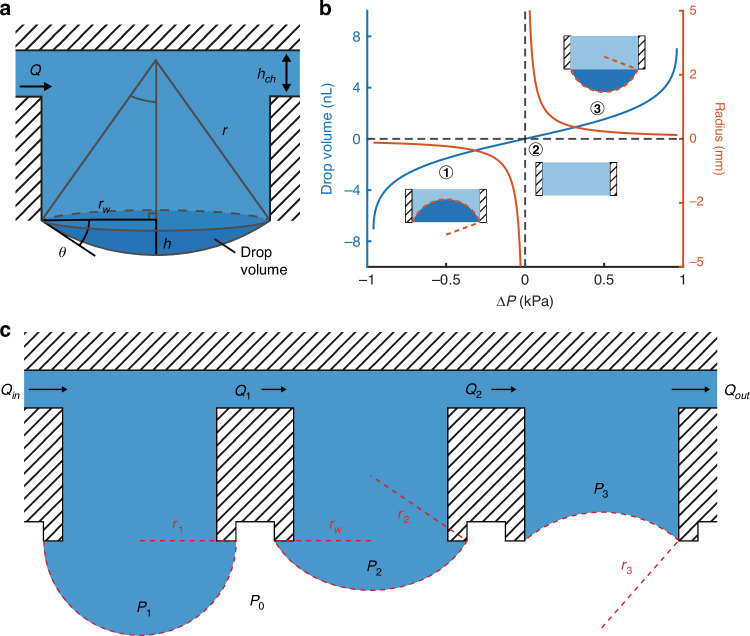
Hanging drop network principles. **a** A schematic of a hanging drop with critical dimensions and the drop volume in dark blue. **b** A graph of the relationship between drop volume, radius, and pressure for convex and concave drops (r_w_ = 150 µm). **c** An illustrative schematic of a multi-drop network during active perfusion

At low volumes, the hanging drop takes the form of a convex spherical cap, defined by the sphere radius ($r$), cap base radius (${r}_{w}$), height of the spherical cap at the center of the cap base ($h$), and angle between the surface tangent and cap base at the cap edge ($\theta$) (Fig. 1a). The volume is typically defined as:

3 $$V=\frac{\pi }{3}{h}^{2}\left(3r-h\right)$$

When working with the Young–Laplace equation, it is most useful to have the volume purely in terms of $r$ and ${r}_{w}$. To obtain this, we can substitute $h=r-\sqrt{{r}^{2}-{{r}_{w}}^{2}}$ in Eq. (3) to obtain:

4 $$V=\frac{\pi }{3}\left(2{r}^{3}-\left(2{r}^{2}+{{r}_{w}}^{2}\right)\sqrt{{r}^{2}-{{r}_{w}}^{2}}\right)$$

When the pressure inside the well is greater than the ambient pressure and the droplet is convex (Fig. 1b, case 3), we will consider the radius as positive, with a minimum value ${r}_{w}$. As the pressure decreases to zero, the droplet becomes flat (Fig. 1b, case 2), and the radius increases to its maximum value ($r=\infty$). For negative pressures, the droplet becomes concave with the interior of the spherical cap occupied by air (Fig. 1b, case 1). Because the volume of the spherical cap is now subtracted from the total well volume, the droplet volume is considered negative—this case is also visualized in the third well of Fig. 1c. We will consider the radius of a concave drop as negative because it results in a well pressure that is lower than the ambient pressure via the Young–Laplace equation. This allows us to define the volume of the droplet over the relevant range as:

5 $$V=\left\{\begin{array}{ll}\frac{\pi }{3}\left(2{r}^{3}-\left(2{r}^{2}+{{r}_{w}}^{2}\right)\sqrt{{r}^{2}-{{r}_{w}}^{2}}\right),{r}_{w}\le r < \infty \,\\ -\frac{\pi }{3}\left(2{\rm{|}}r{{\rm{|}}}^{3}-\left(2{r}^{2}+{{r}_{w}}^{2}\right)\sqrt{{r}^{2}-{{r}_{w}}^{2}}\right),-{r}_{w}\ge -r > -\infty \end{array}\right.$$

At high volumes, the center of curvature extends below the well, and the drop bulges into the form of a sphere with a spherical cap removed. This case (θ > 90°) will not be of interest in systems with connected hanging drops because the system will not be stable.

### Multiple drop system principle

For interconnected drops, we must consider the relationship between the pressures in multiple wells. Two adjacent wells with hanging drops have pressures ${P}_{1}={2\sigma /r}_{1}$ and ${P}_{2}={2\sigma /r}_{2}$, as in Fig. 1c. Because all external pressures are the same, we have reduced $\Delta P$ to $P$ for simplicity. The fixed radius of the well is denoted as ${r}_{w}$ and considered constant because the drop is pinned by features much smaller than the radius. Gravity is neglected, so the pressure within the well is considered uniform. The volumetric flow (${Q}_{1}$) between the two wells separated by a channel of fluidic resistance ${R}_{1}$ is then:

6 $${Q}_{1}=\left({P}_{1}-{P}_{2}\right)/{R}_{1}=\frac{2\sigma }{{R}_{1}}\left(\frac{1}{{{\rm{r}}}_{1}}-\frac{1}{{r}_{2}}\right)=\frac{2\sigma }{{R}_{1}}\left(\frac{{r}_{2}-{r}_{1}}{{r}_{1}{r}_{2}}\right)$$

For both convex and concave cases, an increase in volume in a single drop will result in a decrease in radius and an increase in pressure. This causes a pressure difference between the drops and results in flow into the drop with a lower volume, according to Eq. (6). Once the volume of any drop increases past $r={r}_{w}\,(\theta \,>\, 90^{\circ}\,)$, it will become unstable. As the volume increases, the radius of curvature now increases and pressure decreases. As a result, fluid will flow from the adjacent well at an increasing rate until the system destabilizes.

The principle of a pressure gradient driven by a difference in droplet size has been used for passive pumping through microfluidic devices^24^. It was similarly the driving principle behind media exchange in the two-drop networks demonstrated by de Groot et al. and the integrated pump by Yazdi et al.^18,14^.

### Drop pinning

**Fig. 2:**
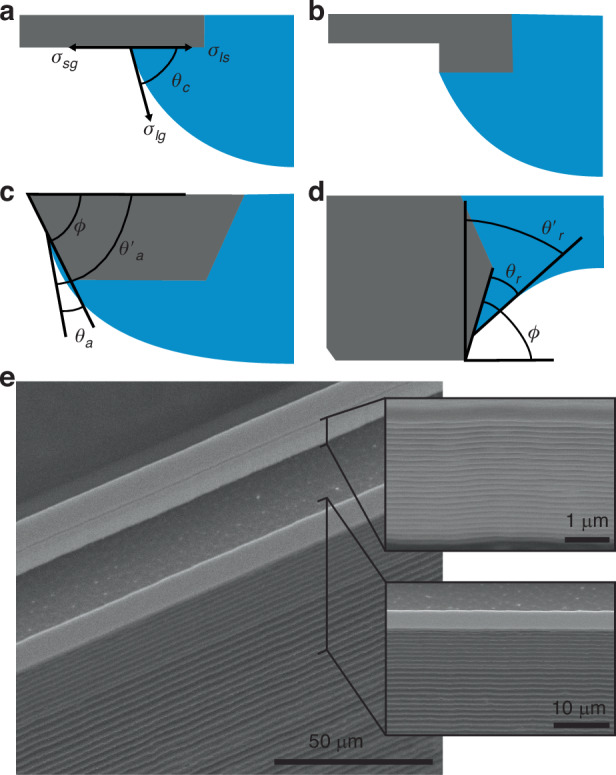
Hanging drop pinning. **a** A diagram of a hanging drop and the resultant contact angle on an unpinned surface. **b** A droplet pinned by a surface feature with sharp edges. Diagrams showing the effective contact angle as the droplet is pinned by the **c** outer edge and **d** inner edge of the pinning features. **e** Scanning electron microscope images showing the pinning features of the fabricated devices, with detailed images of the inner and outer surfaces

Consideration of the drop boundary is also critical to designing hanging drop systems. The drop boundary is described by Young’s equation^21^:

7 $${\sigma }_{{sg}}={\sigma }_{{sl}}+{\sigma }_{{lg}}\cos {{\rm{\theta }}}_{{\rm{C}}}$$

where ${\sigma }_{{sg}}$, ${\sigma }_{{sl}}$, and ${\sigma }_{\mathrm{lg}}$ are the surface tension of the solid-gas, solid-liquid, and liquid-gas interfaces, respectively, and ${{\rm{\theta }}}_{{\rm{C}}}$ is the resultant contact angle (Fig. 2a). In practice, drops often exhibit some contact angle hysteresis, and distinct advancing (${{\rm{\theta }}}_{{\rm{a}}}$) and receding (${{\rm{\theta }}}_{{\rm{r}}}$) contact angles can be measured. For a convex hanging drop on a flat surface, angle $\theta$ is necessarily the contact angle described by Young’s equation; an increase in drop volume tends to increase $\theta$, resulting in the drop spreading across the surface to maintain a consistent contact angle. This is generally an unacceptable effect for hanging drop arrays, as spreading drops will eventually meet and merge. This is especially a concern when the contact angle is low, and even miniscule volume increases can cause rapid spreading of drops.

To form an array of droplets with a consistent footprint, pinning features must be introduced. Generally, two approaches have been used. In the first, sharp edges are patterned around the desired drop perimeter (Fig. 2b). The drop boundary will remain pinned on the edge until the volume is large enough that the advancing contact angle can be formed with the feature sidewall and the boundary extends beyond the edge (Fig. 2c). Because of the angle of the sidewall ($\phi$), a new effective contact angle for the drop is formed:

8 $${\theta }_{a}^{{\prime} }={\theta }_{a}+\phi$$

resulting in pinning. This holds until expansion results in a $\theta$ is greater than ${\theta }_{a}^{{\prime} }$ and the drop boundary moves down the sloped surface^25^. Microfabricated rings with heights greater than ~2 µm have been shown to demonstrate effective pinning^26^. Similar features are utilized in macroscale plate format hanging drop arrays and the majority of previous hanging drop arrays, although usually on the scale of hundreds of microns (Supplementary Information Table 1)^27^.

In the devices fabricated here, a sharp edge also serves to pin the drop as it recedes into a convex shape. Like the case of the advancing edge, the drop will remain pinned on the inner edge until the receding contact angle is reached with the sidewall surface. After this, an effective receding angle will be formed (Fig. 2d):

9 $${\theta }_{r}^{{\prime} }=\left(90^{\circ} -\phi \right)+{\theta }_{r}$$

In the case of the scalloped surface resulting from deep reactive ion etching, describing any further recession requires consideration of rough capillary rise (Fig. 2e)^28^.

The other common method for droplet pinning involves selective modification of the surface chemistry^29–32^. By providing a surface with a large contact angle around the feature perimeter, the droplet can be effectively pinned at the boundary of this patterned surface. However, this surface modification can inhibit the wetting of the array if it extends to the interior surfaces of the device. Therefore, selective surface modification must be achieved during fabrication. The choice between the two methods, therefore, depends mainly on the limitations imposed by the fabrication processes used for the device.

A preliminary version of the hanging drop microarray that does not include retaining features is shown in Fig. S1. Without the retaining features, the droplets merge easily as the contact line creeps along the device surface toward adjacent wells.

### Analytical model (steady state)

**Fig. 3:**
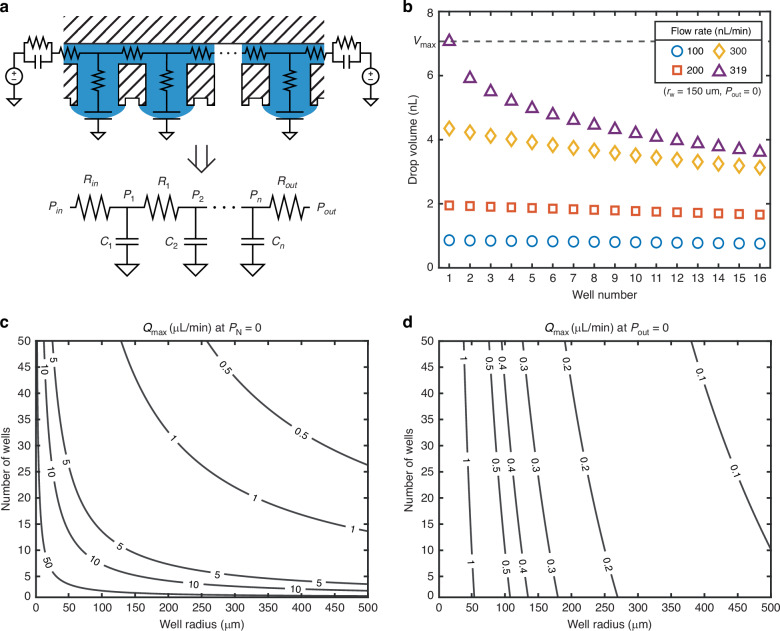
Analytical modeling of a series hanging drop network. **a** Initial and simplified schematics of the electronic-hydraulic analog for a series of hanging drops in an array. **b** Modeling of the drop volume across a range of flow rates. Contour plots are generated to guide the selection of device parameters based on the desired flow rate in cases where **c** the last well is held at atmospheric pressure and **d** the outlet is held at atmospheric pressure

Based on the principles described in the previous sections, we can develop a circuit analog model from the network of wells (capacitors) and channels (resistors). A detailed version is presented superimposed on the device schematic (Fig. 3a). In the case of the devices fabricated here, the resistance within the well is negligible relative to the channel resistance and can be ignored. Because the devices are formed in rigid silicon and glass (unlike previous networks in PDMS), the compliance of the device itself does not need to be considered. And while the tubing capacitance is often considered in complex models, it is much lower than the capacitance of the individual wells in this system. This allows for considerable simplification of the circuit diagram.

With this model, we can make some useful analytical statements about the steady-state behavior. First, we will assume that each resistance between wells is the same (${R}_{{ch}}$) but distinct from the resistance at the inlet and outlet (${R}_{{{\mathrm{out}}}}$). At steady state, where no volume change is occurring, the flow rate ($Q$) from each well must also be the same as at the inlet and outlet. By adding the pressure drop after a particular well to the outlet pressure, we can find the pressure in the nth well:

10 $${P}_{n}={P}_{n+1}+Q{R}_{{ch}}={P}_{{{{out}}}}+Q{R}_{{{{out}}}}+\left(N-n\right)Q{R}_{{ch}}$$

where $N$ is the number of wells and ${P}_{{{{out}}}}$ is the pressure at the outlet. Using this relationship with Eq. (5) and the Young–Laplace equation, we can find the steady-state volumes of a series of wells over a range of flow rates (Fig. 3b). Notably, at high flow rates, the nonlinear relationship between the volume and pressure can be observed. The system will become unstable if ${P}_{1}$ exceeds its maximum value $\left({P}_{max}=2\sigma /{r}_{w}\right)$, which is based on the pinned radius of the well, ${r}_{w}$. While the system will not immediately become unstable if a well pressure falls below $-{P}_{\max }$, it will depart from its normal, predictable behavior; therefore, -${P}_{\max }$ is considered the minimum pressure in a well. Under these conditions, the absolute maximum flow rate is:

11 $${Q}_{{max} ,{{{abs}}}}=\frac{2{P}_{max}}{\left(N-1\right){R}_{{ch}}}$$

For practical control of the system, it is more useful to consider the maximum rate as a function of the outlet pressure:

12 $${Q}_{max }=\frac{{P}_{max }-{P}_{{{{out}}}}}{{R}_{{{{out}}}}+\left(N-1\right){R}_{{ch}}}$$

Meanwhile, the maximum number of wells is:

13 $${N}_{\max }=1+\left\lfloor \frac{{P}_{\max }}{Q{R}_{{ch}}}-\frac{{P}_{{out}}}{Q{R}_{{ch}}}-\frac{{R}_{{{\mathrm{out}}}}}{{R}_{{ch}}}\right\rfloor$$

which can only be defined for a certain flow rate, $Q$.

If we configure the system such that the final well in the series has a pressure equal to atmospheric (requiring an outlet pressure below atmospheric for positive flow), these can be simplified to:

14 $${N}_{max}=1+\left\lfloor \frac{{P}_{max}}{Q{R}_{{ch}}}\right\rfloor$$

15 $${Q}_{max}=\frac{{P}_{max}}{\left(N-1\right){R}_{{ch}}}$$

These equations supply a starting point for the design of connected hanging drop arrays. For the devices fabricated here, a channel height of 10 µm was chosen to reduce the possibility of cell movement between wells, and a layout with a channel width of 100 µm and length of 20 µm was selected guided by fabrication considerations. Based on our group’s previous optimization for cell culture by well size, we focused on diameters from 100 to 500 µm^20^. After approximating the channel resistances, a chart like those in Fig. 3c-d can be generated from Eqs. (12–15) to select the number of wells and well radius based on the desired flow rate. For our demonstration, we chose a well size equivalent to a radius of about 150 µm and 16 series wells, constraining the flow rate to between 1 and 5 µL/min under optimal conditions (Fig. 3c). However, the flow rate is constrained significantly if the outlet is held at atmospheric pressure, demonstrating the critical role of the outlet resistance (Fig. 3d).

This framework for the selection of key parameters can be applied on any scale from nano- to millimeters. However, an important observation is that as a network length scale, $l$, is reduced (reducing both channel radius and drop radius proportionally), the resistance will tend to increase with ${l}^{4}$ while the well radius decreases only with $l$. Thus, ${N}_{\max }$ and ${Q}_{\max }$ will be increasingly constrained at smaller scales. Supplementary Information Note 1 contains the full nondimensionalization of the pressure and volume equations for characterizing these systems across a broad range of size scales.

### Numerical model (time-dependent)

In the electronic-hydraulic analogy, the hanging drop can be modeled as a capacitor because it reacts to oppose changes in pressure. However, its response is nonlinear. The constitutive equations applicable across concave and convex hanging drops are derived in Supplementary Information Note 2 as:

16 $$V\left(\hat{p}\right)=\left\{\begin{array}{rl}\frac{\pi{{r}_{w}}^{3}}{3{\hat{p}}^{3}}\left(2-\left(2+{\hat{p}}^{2}\right)\sqrt{1-{\hat{p}}^{2}}\right),& 0 \,< \, \left|\hat{p}\right|\le 1\,\\ 0,&\hat{p}=0\end{array}\right.$$

and

17 $$Q=\frac{{dV}}{{dt}}=C\frac{{dP}}{{dt}}$$

with

18 $$C={V}^{{\prime}}\left(\hat{p}\right)=\left\{\begin{array}{rcl}\frac{\pi {{r}_{w}}^{4}}{2\sigma {\hat{p}}^{4}}\left(\frac{2-{\hat{p}}^{2}}{\sqrt{1-{\hat{p}}^{2}}}-2\right),&0\, < \, \left|\hat{p}\right| < 1\\ \frac{\pi {r}_{w}^{4}}{8\sigma },&\hat{p}=0\end{array}\right.$$

where $Q$ is the volumetric flow rate, $V$ is the volume, and $C$ is the compliance (analogous to current, charge, and capacitance, respectively). ${r}_{w}$ denotes the pinned radius of the well which is fixed by the device geometry. $\hat{p}$ is a unitless parameter defined as $\frac{P}{{P}_{\max }}$, with ${P}_{\max }=\frac{2\sigma }{{r}_{w}}$ describing the pressure at which a drop becomes unstable. This value can be treated like the breakdown voltage of a capacitor.

**Fig. 4:**
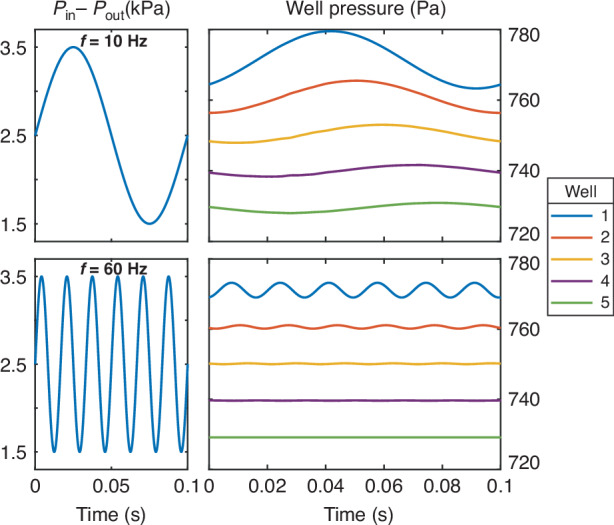
Time-dependent modeling of network dynamics. Time-dependent modeling of the fabricated devices with variable input frequency shows the increased attenuation of high-frequency pressure waves

We can create a system of differential equations for the hanging drop network (Supplementary Information Note 3), resulting in the relationship:

19 $${P}_{n}^{{\prime} }\left(t\right)=\frac{{P}_{n-1}\left(t\right)-\frac{{P}_{n}\left(t\right)-{P}_{n+1}\left(t\right)}{{R}_{n}}{R}_{n-1}-{P}_{n}\left(t\right)}{{V}^{{\prime} }\left({P}_{n}\left(t\right)\right){R}_{n-1}}$$

where ${P}_{n}\left(t\right)$, ${P}_{n}^{{\prime} }\left(t\right)$, and ${R}_{n}$ are the pressure, derivative of pressure with respect to time, and channel resistance of the nth drop and corresponding channel, respectively. The well pressures can then be solved numerically with a first-order ordinary differential equation solver by specifying the device geometry, the initial pressure for each well (${P}_{n}\left(0\right))$, and the pressure at the inlet and outlet (${P}_{0}\left(t\right),{P}_{N+1}\left(t\right))$ over time. An example of the modeled response for a five-well system is given in Fig. 4, with more examples in Fig. S2.

In these responses, the pressure change introduced by the 10 Hz sine wave is visible throughout all wells, while the 60 Hz sine wave is strongly attenuated after the first well. This is because the device effectively acts as a multi-stage low-pass filter. As such, we can consider the response of a single stage using a time constant, $\tau ={R}_{{ch}}{C}_{{{\mathrm{well}}}}$. This characterization can be used only broadly because the well response is nonlinear. However, it can provide an approximation of the cutoff frequency ($1/2\pi \tau$) below which a certain design will effectively attenuate pressure variations. This is particularly significant to hanging drop networks because they become unstable in response to much smaller input pressure variations than typical closed microfluidic systems. In this type of system, the time constant is also related to the time it takes for a pressure wave to propagate through the system. As a result, the time constant also relates to the length of a pressure pulse that the system can tolerate. Notably, the resistance of a channel with a critical dimension $l$ scales with ${l}^{-4}$ while the compliance of a single drop scales with ${l}^{4}$. Consequently, scaling both components at the same rate maintains the time constant and should result in a similar frequency response. However, the compliance of the surrounding components should also be considered. Because the compliance of a droplet with a 150 µm radius is typically between 10 and 100 times higher than the tubing used in this system (Supplementary Information Note 4), this modeling is expected to match the system response well. For much smaller hanging drops, however, the compliance might be negligible compared to the surrounding system.

Our approach contrasts with the two-drop modeling demonstrated by de Groot et al., which utilized an iterative numerical method for solving the shape of the droplet surfaces^18,33^. This type of numerical approach is better suited to large pendant droplets where gravity is a significant consideration. However, it requires a more involved numerical approach that may be difficult to adapt to large or complex networks. Rousset et al. similarly modeled droplets close to a Bond number of one, where the behavior is primarily constrained by gravitational effects and the failure mode is a “drop crash” when the capillary pressure of the feature is exceeded^16^. This modeling also did not account for droplet behavior at negative pressures, which can occur routinely in nanoliter-scale droplets.

### CFD flow modeling

**Fig. 5:**
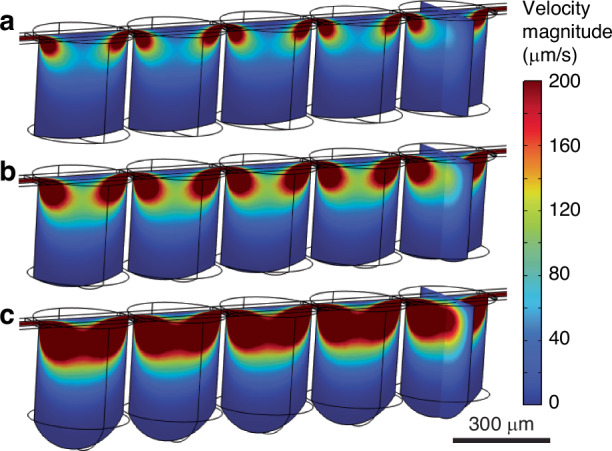
Computational fluid dynamics modeling. Fluid velocity magnitude is shown through five series wells (r_w_ = 150 µm, h_channel_ = 10 µm) with flow of **a** five, **b** ten, and **c** twenty well volumes per minute

To further understand flow conditions within the well, a five-well device was simulated in COMSOL. A no-slip condition was applied on all solid device surfaces and a slip condition on the fluid-fluid interface. The pressure at the outlet was set to atmospheric and the flow at the inlet was varied. Flow rates were chosen such that five, ten, and twenty well volumes would be replaced per minute (106, 212, and 424 nL/min, respectively). Before each simulation, the drop volumes were updated based on the well pressure, in line with our analytical model. The modeled results are shown in Fig. 5.

In our configuration with fully enclosed channels, high-velocity flow is confined to the upper portion of the well, even at flow rates near the system limitations. This is consistent even at a channel height of 100 µm (Fig. S3). Further, the change in drop volume because of the internal pressure does not appear to alter the steady-state flow profile. This contrasts with the results for arrays with a continuous fluid-fluid interface, where the highest flow is along the interface, making displacement of cells a significant consideration^13,17^. In a closed platform like ours, higher flow rates should be possible without sweeping cells away from the liquid-air interface. This modeling also indicates that the exchange of solutes between the perfused flow and the drop is likely to occur primarily by diffusion. While the diffusion distance in our devices is predicted to be less than 200 µm, resulting in rapid diffusion, this is a key consideration for the design. Future work should use coupled flow and solute transport modeling to optimize cell culture conditions within the hanging drops. The potential interaction between wells in series is a particularly important consideration because the transfer of soluble factors between wells may influence cell behavior. Because the diffusion distance depends on the height of the well, this may also be an important parameter to optimize diffusive transport to the hanging drops while minimizing shear stress. This flow modeling does not account for flow resulting from temporal drop volume changes or evaporation, which may be significant to solute transport.

### Cell culture considerations

Several significant considerations arise in the case of cell culture. The value of surface tension is significant because of its presence in many of the key analytical predictions. Cell culture medium contains significant electrolyte concentrations known to slightly increase surface tension^34^. However, popular medium formulations also include additives such as fetal bovine serum (FBS), which contain molecules that act as surfactants^35^. For example, the addition of 3% FBS to the cell culture medium has been shown to reduce the surface tension from the near-water 69 to 52 mN/m. Cell culture also requires increased temperature, which acts to further reduce the surface tension. As discussed in Supplementary Information Note 2, the surface tension changes the full pressure range of the system but does not otherwise impact its operation. Therefore, a reduction in surface tension can be compensated for by scaling the pressure values.

Another significant consideration is the availability of soluble media components and dissolved gases. Because the volume of the hanging drop at this scale is on the order of nanoliters, high cell numbers may deplete the media of critical soluble components and accumulate waste byproducts at a high rate. This was the limiting factor for long-term culture in our group’s previous hanging drop platform without fluidic connections^20^. At the same time, rapid evaporation due to the high surface-area-to-volume ratio will result in increased osmolality. Because these effects are more significant in smaller wells, this increasingly necessitates active perfusion at reduced well size. This consideration depends heavily on the cell type and number used, and so should be studied in more detail to determine the minimum necessary perfusion. At any scale, gas exchange is facilitated by the open fluid surface if the device is kept in a culture chamber with optimal gas composition.

Finally, the hanging drop array needs to be composed of biocompatible materials and should facilitate cell seeding and imaging. In the chosen design, all exposed surfaces are covered in silicon dioxide, which is bioinert^36^. To facilitate efficient cell seeding at low cell numbers, as in our previous publication, the array was designed so that cell seeding could occur through the open well surface. Wells were designed as squares to fully utilize the chip surface area and reduce cell loss during this process. Fabrication was updated from our previous platform to provide fully transparent wells that facilitate live cell imaging. A thin glass wafer (175 µm thickness) was chosen to match the thickness of a typical cover slip and ensure compatibility with a variety of high-magnification live cell imaging systems.

### Device fabrication

**Fig. 6:**
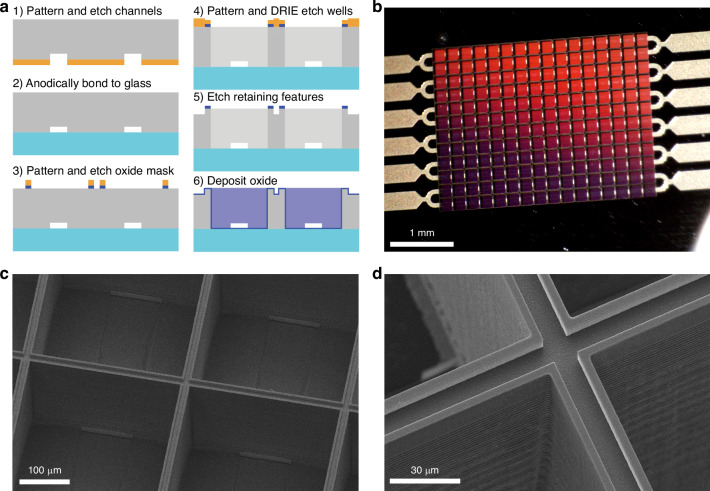
Fabrication of nanoliter-volume hanging drop arrays. **a** Fabrication schematic for the transparent, nanoliter-volume hanging drop array with embedded channels and surface retaining features. **b** An optical image of a completed 12 × 16 array with channels visible between the wells. **c** A scanning electron microscope image of the same device with retention features and embedded channels visible. **d** An enlarged image of the droplet retention features

The device fabrication is summarized in Fig. 6a, and a detailed protocol is provided in the Supplementary Information. Figure 6b–d highlight key features of the fabricated devices. Three etching layers were used to define the channels, wells/ports, and retaining features. An anodically bonded glass wafer was introduced to allow for imaging through the platform. Another key benefit of the purely silicon and glass construction of this device is that it can be piranha cleaned and reused repeatedly, which is not possible for the majority of demonstrated hanging drop arrays.

Two key challenges were encountered in the fabrication of this device. First, notching defects occurred when exposing the glass layer at the end of the deep etching step. This was mitigated by switching from a typical high-frequency (13.56 MHz) etch to a gentler but slower low-frequency (380 kHz) etch. Second, damage occurred to the retaining features when an oxide mask was not used as specified in final fabrication protocol. Additional information is included in Supplementary Information Note 5.

### Interface and system

**Fig. 7:**
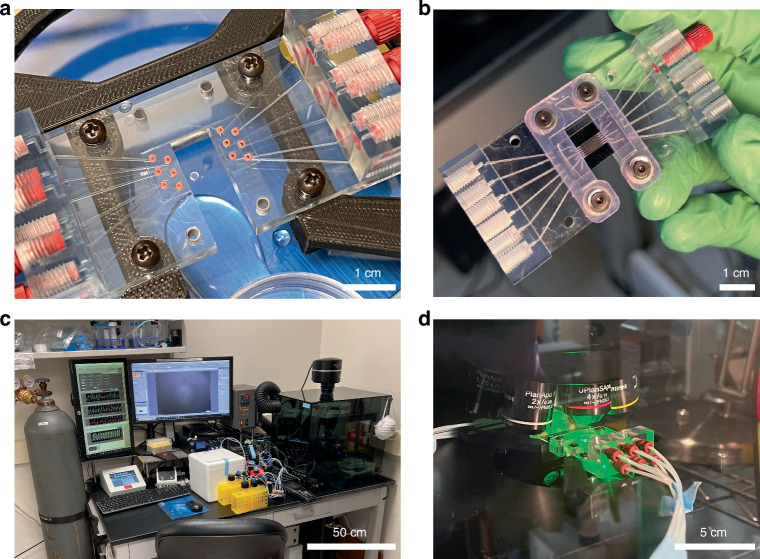
Images of the custom interface and experimental apparatus. **a** A top view of the interface with the O-rings visible before insertion of the device. **b** The chip after being secured in the interface. **c** The experimental chamber with all supporting systems. **d** The device being imaged during an experiment

Because of the small scale of the device, a custom interface was necessary to provide a fluidic connection. 3D printing the interface in a clear, biocompatible resin was found to be optimal because it allowed for high resolution, reasonable design freedom, and easy visualization of flow in the interface (Fig. 7a). A top plate was used to provide adequate clamping force (Fig. 7b). This interface allowed for quick connection of the device to standard 1/16” PTFE tubing via 10–32 threaded connectors.

To support this device, a custom chamber was designed around an inverted microscope capable of brightfield and fluorescent imaging (Fig. 7c). The device and interface can be seen within the chamber during imaging in Fig. 7d. Because hanging drops below the millimeter scale are especially sensitive to environmental perturbations, an enclosed chamber is necessary to consistently provide optimal temperature and humidity. A chamber is also necessary to provide a suitable CO_2_ concentration and preserve sterile conditions for cell culture experiments. A detailed diagram of the system is presented in Fig. S4. A pressure-based pump was used for the fluid control. This was selected over syringe pumps or other flow-controlled pumps because pressure-based control will naturally compensate for evaporative losses (via the changes in droplet Laplace pressure) and is less likely than a volumetric pump to destabilize the chip during a flow disturbance.

### Device validation

**Fig. 8:**
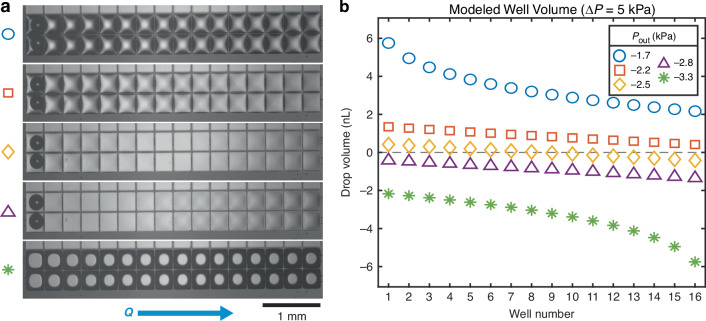
Performance of the hanging drop network over its operational pressure range. **a** On-chip images of water flow (in the direction of the blue arrow) under progressively decreasing outlet pressures. A single inlet/outlet is split into two paired columns in the device. **b** Modeled results for the same scenario, showing general agreement with the predicted behavior

In practical tests of the platform, the simplest flow control scheme is achieved by fixing the pressure difference between the inlet or outlet based on the desired flow rate (informed by the analytical model). Once the system is stable, both pressures can be adjusted in parallel until the desired well volume is achieved. Typical flow under this strategy is shown in Fig. 8a. To demonstrate the relationships predicted by the analytical model, the pressure difference was fixed at 5 kPa and the outlet pressure was swept over a range of values. Under similar flow rates (~900 nL/min), the droplets were observed to progress from fully convex to fully concave. The predicted hanging drop volumes can be seen to vary roughly in line with the predictions of the analytical model (Fig. 8b). Upon reaching pressures well below atmospheric pressure, fluid was aspirated from the wells, resulting in the system drying out. Clogging was also sometimes observed at low outlet pressures, likely due to bubble formation (or expansion of existing bubbles formed during system priming)^37^. This may be mitigated by maintaining pressures in the device above atmospheric pressure to inhibit bubble expansion.

While this device is designed to run multiple channels in parallel, it is challenging to run multiple channels from the same pressure source. This seems to be because of minor differences in channel resistance that make it difficult to achieve exactly the same drop volumes in parallel. This could be mitigated by using separate pressure channels for each inlet, but the cost of pressure-based pumps is significant in this control scheme. To demonstrate the use of all channels in parallel, an image of an array with all wells filled under low flow (<100 nL/min) is shown in Fig. S5.

## Conclusion

In this paper, we approached the design of fluidically connected hanging drop networks at a size scale below what has been previously reported. We constructed an analytical model from the physical principles of hanging drops at the microscale and used this to provide design guidelines. We developed a simple time-dependent model based on droplet compliance that can be adapted to different configurations of nanoliter-scale hanging drop networks. We included computational fluid dynamics modeling of flow within wells, which varies significantly from previous open-channel designs. We described the steps that were taken to fabricate the device and surrounding system. Finally, we demonstrated the basic operation of the first hanging drop network with nanoliter-volume droplets.

Operating a network of this size comes with significant challenges inherent to the scale, including increased evaporation and channel resistance. Although smaller droplets are more resistant to pressure variation, volume fluctuations on the order of nanoliters are enough to collapse the system. Many aspects of hanging drop network performance on this scale still need to be studied, especially concerning cell culture. Hanging drop networks at this scale require frequent or active perfusion beyond that of larger platforms, which introduces challenges. However, smaller platforms hold the potential for more rapid spheroid formation or spheroid formation from smaller cell numbers. Further analytical or numerical models should be developed to account for the diffusion of solutes relevant to cell culture and the effect of evaporation on flow within the wells, which may be significant at this size scale.

## Materials and methods

### Modeling

The full derivation of the hanging drop constitutive equations can be found in Supplementary Information Note 2. A detailed description of the analytical modeling approach, including MATLAB code, can be found in Supplementary Information Note 3. Steady-state modeling calculations and plots were generated in MATLAB. Computational fluid dynamics (CFD) simulations were carried out in COMSOL Multiphysics® version 6.2. Before simulation, well volumes were calculated using the steady-state analytical model described in this report, and the parametric geometry was adjusted accordingly.

### Device fabrication

A detailed fabrication protocol can be found in the Supplementary Information Experimental Details. A description of the key steps is provided here. First, channels were etched to ~10 µm depth in a 300-µm-thick silicon wafer. The wafer was dry oxidized to form a roughly 200-nm oxide layer and anodically bonded to a 175-µm-thick borofloat glass wafer to enclose the channels. A buffered oxide etch was used to selectively remove the oxide from the exposed side of the silicon wafer. Well and port features were etched entirely through the silicon wafer using deep reactive ion etching. Finally, approximately 5-µm-deep trenches were etched on the silicon surface to define the retaining features. A final etch was used to remove any remaining oxide and silicon grass. A 200-nm-thick oxide layer was deposited over the exposed silicon using plasma-enhanced chemical vapor deposition. The wafer was split into individual devices by snapping along etched borders to yield devices of a consistent size. Immediately before wetting with fluid for experiments, devices were treated with O_2_ plasma in a barrel plasma etcher (Pico, Diener Electronic, Ebhausen, Germany) for 5 min at 150 W to clean the surface and render it hydrophilic. Devices were wetted from the inlet side to avoid trapped air.

### System fabrication

An interface utilizing nitrile O-rings (Item #5747, Precision Associates Inc., Minneapolis, MN, USA) for sealing to the twelve device ports was designed and printed in biocompatible clear resin (Somos WaterShed XC11122; fabricated by Proto Labs Inc., Maple Plain, MN, USA) to convert from the fabricated chip to standard 10–32 microfluidic fittings. Two channels of a pressure-based pump (OB1 Mk3+, Elveflow, Paris, France) were used to provide pressure on the inlet and outlet side of the device. The flow was monitored with a thermal mass flow sensor (MFS2, Elveflow). When multiple channels were run in parallel, micro-metering valves (IDEX Health & Science, Oak Harbor, WA, USA) were used to manually approximate the same fluidic resistance in multiple channels. A custom acrylic chamber was fabricated around an upright microscope (Olympus BX63, Evident, Tokyo, Japan) to allow for control of temperature and gas flow along with humidification.

## Supplementary information

Supplementary Information
