# Supplementary material for: On the design and fabrication of nanoliter-volume hanging drop networks
Source: Microsyst Nanoeng. 2024 Oct 16;10:147. doi: 10.1038/s41378-024-00788-0 (PMC11484691; doi:10.1038/s41378-024-00788-0)
Supplement: Supplementary file 1 — Supplementary Information [file 41378_2024_788_MOESM1_ESM.docx]

Supplementary Information

On the Design and Fabrication of Nanoliter-Volume Hanging Drop Networks

Matthew Wester^1,2^, Jongwon Lim^1,2^, Liliana Khaertdinova^1,2^, Sriya Darsi^1,2^, Neel Donthamsetti^1,2^, Glennys Mensing^2^, George Vasmatzis^3,4^, Panos Anastasiadis^4,5^, Enrique Valera^1,2,6,7^, Rashid Bashir*^,1,2,4,6,7,8,9,10,11,12^

^1^Department of Bioengineering, University of Illinois Urbana-Champaign, Urbana, IL 61801, USA

^2^Nick Holonyak Jr. Micro and Nanotechnology Laboratory, University of Illinois Urbana-Champaign, Urbana, IL 61801, USA.

^3^Center for Individualized Medicine, Mayo Clinic, Rochester, MN 55905, USA

^4^Mayo-Illinois Alliance for Technology-Based Healthcare, Urbana, IL 61801, USA.

^5^Department of Cancer Biology, Mayo Clinic, Jacksonville, FL 32224, USA

^6^Carl R. Woese Institute for Genomic Biology, University of Illinois at Urbana-Champaign, Urbana, Illinois 61801, USA.

^7^Biomedical Research Center, Carle Foundation Hospital, Urbana, IL 61801, USA.

^8^Cancer Center at Illinois, University of Illinois at Urbana-Champaign, Urbana, IL 61801, USA.

^9^Department of Biomedical and Translation Science, Carle Illinois College of Medicine, University of Illinois at Urbana-Champaign, Urbana, IL 61801, USA.

^10^Department of Electrical and Computer Engineering, University of Illinois at Urbana-Champaign, Urbana, IL 61801, USA.

^11^Department of Mechanical Science and Engineering, University of Illinois at Urbana-Champaign, Urbana, IL 61801, USA.

^12^Department of Materials Science and Engineering, University of Illinois at Urbana-Champaign, Urbana, IL 61801, USA.

**
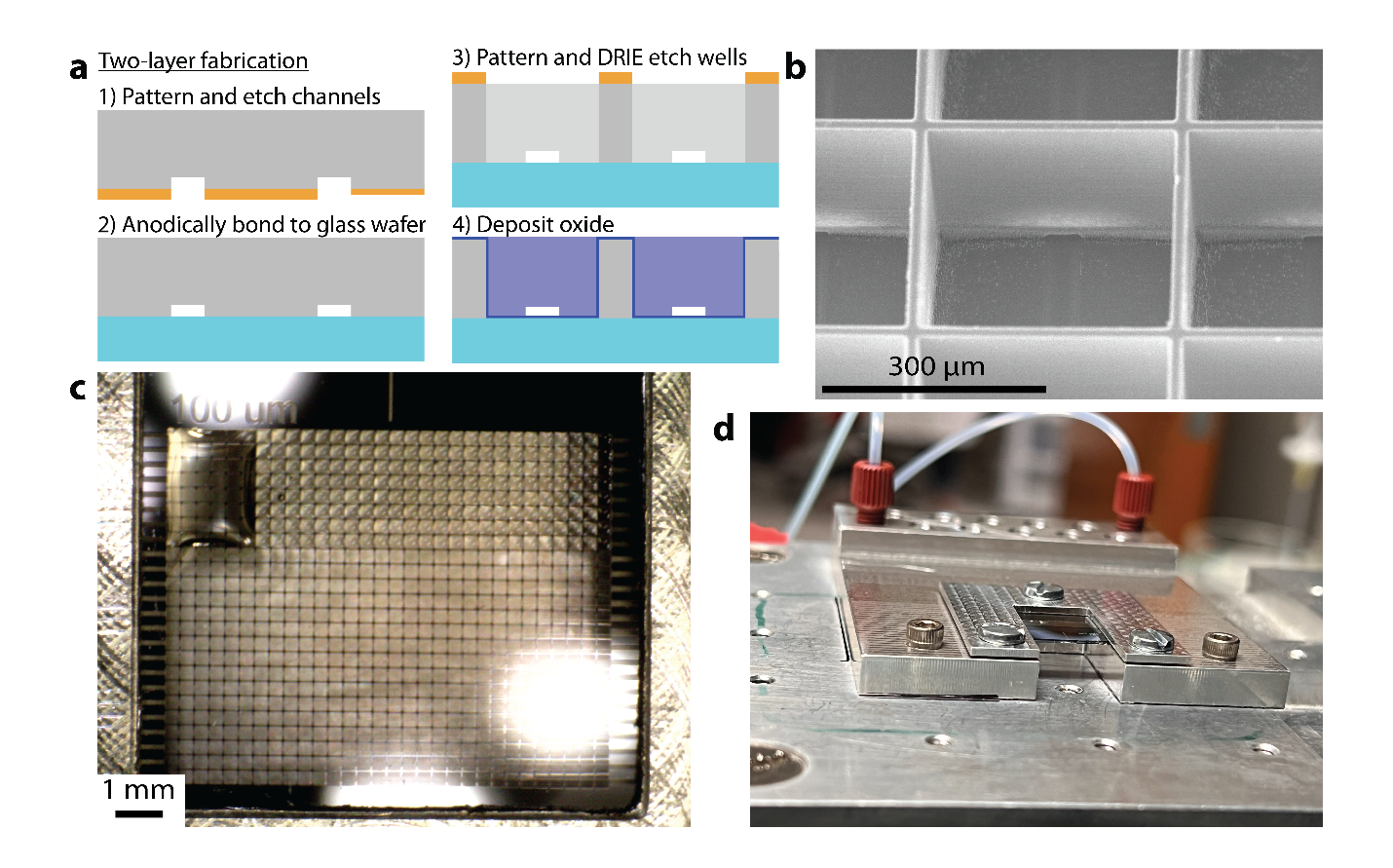
Figure S1.** Preliminary version of the hanging drop array platform. (a) Schematic of an initial fabrication protocol that does not include the retaining features. (b) The scanning electron microscope image shows successful fabrication of the other key features. (c) Individual drops can be seen on the right while drops on the left merge as increasing pressure causes the drops to creep along the surface and merge. (d) An earlier version of the device interface machined in aluminum.

**
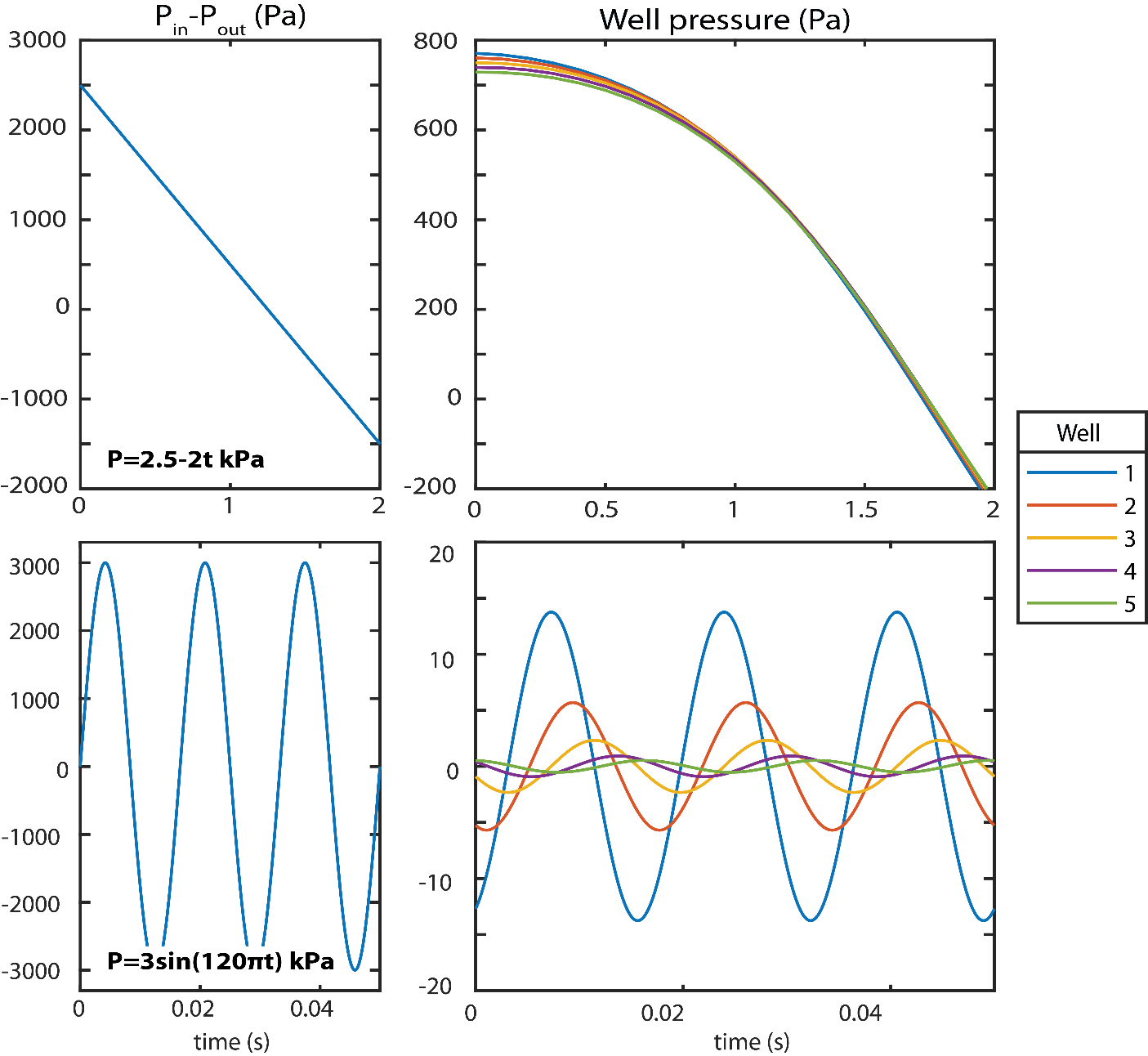
Figure S2.** Time-dependent modeling of the fabricated devices, demonstrating the response to a linear change in pressure and the stability of the model to zero crossings.

**
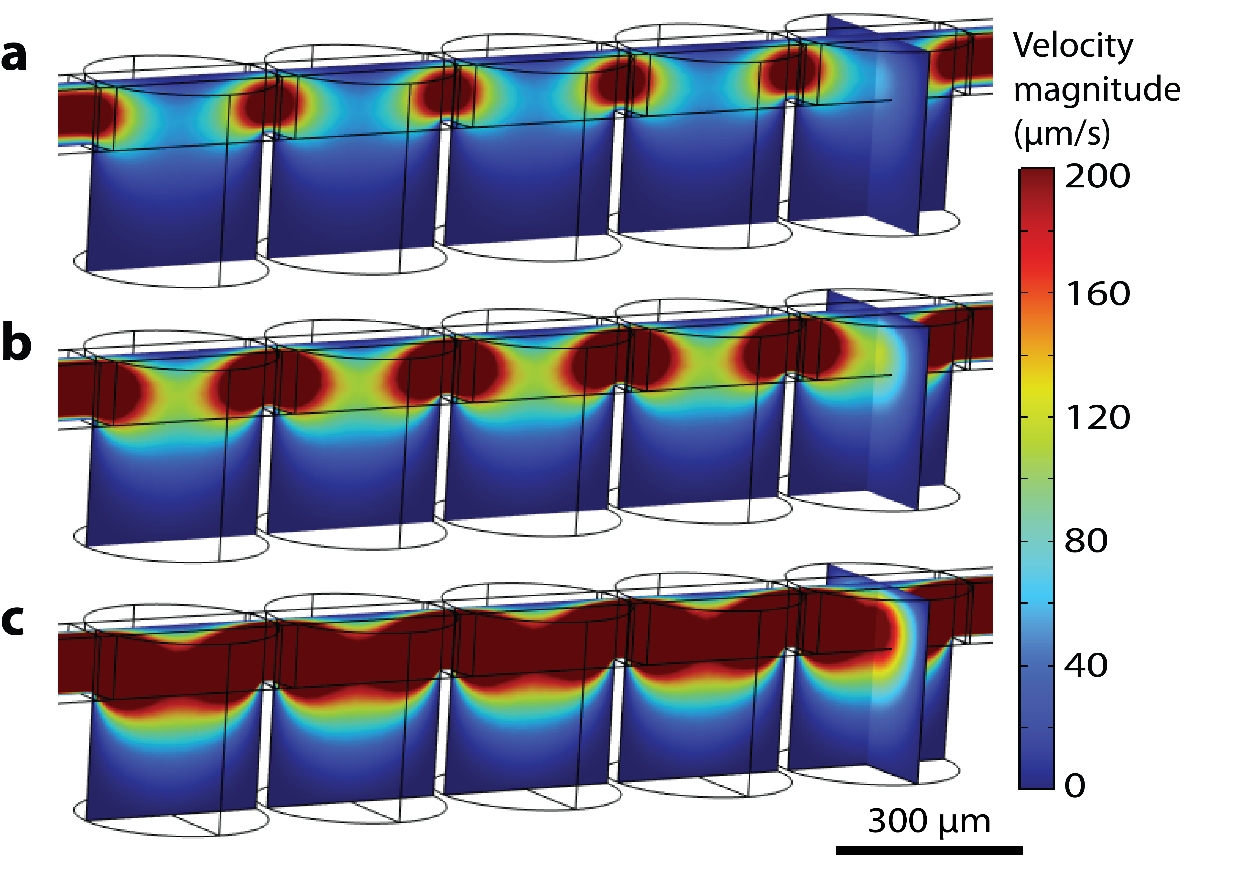
Figure S3.** Fluid velocity is shown through five series wells (r_w_=150 µm, h_channel_=100 µm) with flow of (a) five, (b) ten, and (c) twenty well volumes per minute.

**
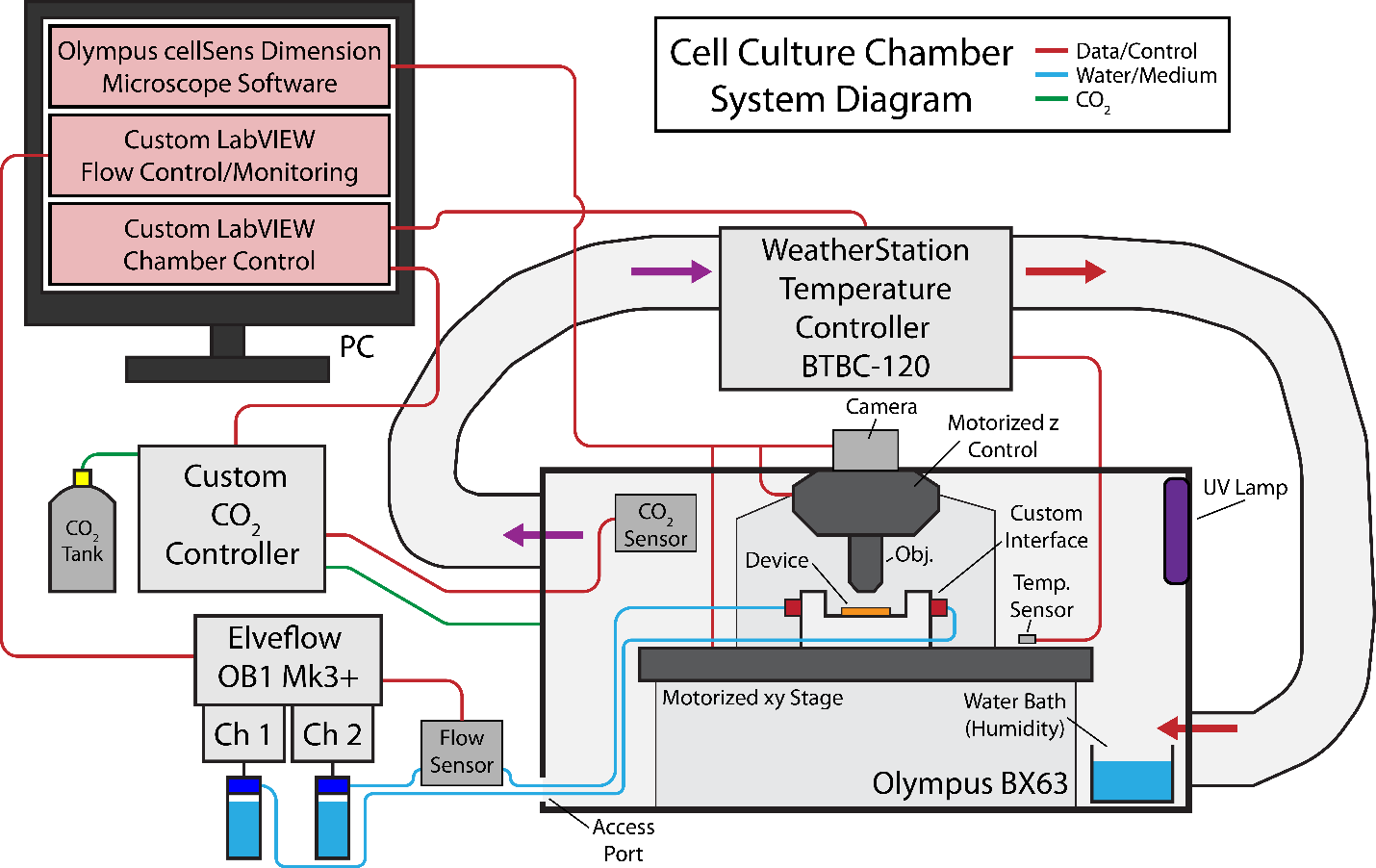
Figure S4.** A system diagram of the environmental/cell culture chamber developed for support of this device, with all major components included.

**
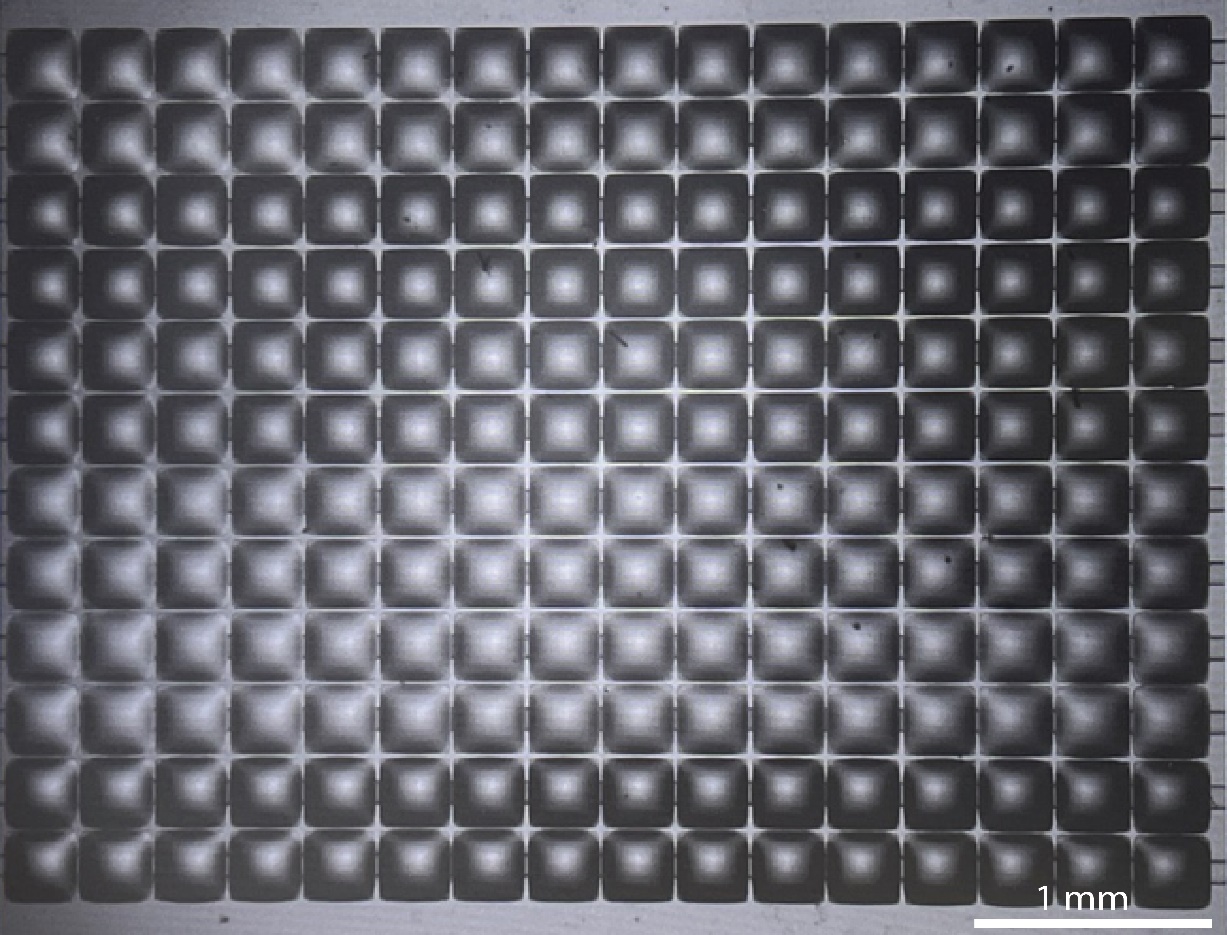
Figure S5.** Well volumes across all channels can be seen to equilibrate under low-flow conditions
(<100 nL/min).

**Note 1**

**Nondimensionalization of key equations**

Starting from Equation (10) in the main text for the pressure in the n^th^ well:

$$\begin{aligned} P_{n}=P_{out}+QR_{out}+\left( N-n \right)QR_{ch}\#\left( S1 \right) \end{aligned}$$

We can define:

$$\begin{aligned} \hat{p}_{n}=\frac{P_{n}}{P_{max}} \mathrm{and}\hat{p}_{out}=\frac{P_{out}}{P_{max}}\#\left( S2,S3 \right) \end{aligned}$$

with $\hat{p}_{n}$ ranging from -1 to 1 ($P_{atm}=0)$and $\hat{p}_{out}$ limited by values that bring the first well above $P_{max}$ or the last well below $-P_{max}$. Additionally, we can nondimensionalize the flow rate by stating:

$$\begin{aligned} \hat{q}=\frac{Q}{Q_{max,abs}}\#\left( S4 \right) \end{aligned}$$

with $\hat{q}$ ranging from 0 to 1. In this case, $Q_{max,abs}$ describes the absolute maximum flow rate, which occurs when the first well is at the maximum possible pressure and the last well is at the minimum possible pressure. This means that:

$$\begin{aligned} P_{1}=P_{max}=P_{out}+Q_{max,abs}R_{out}+\left( N-1 \right)Q_{max,abs}R_{ch}\#\left( S5 \right) \end{aligned}$$

and:

$$\begin{aligned} P_{N}=-P_{max}=P_{out}+Q_{max,abs}R_{out}\#\left( S6 \right) \end{aligned}$$

From this, we can solve:

$$\begin{aligned} Q_{max,abs}=\frac{2P_{max}}{\left( N-1 \right)R_{ch}}\#\left( S7 \right) \end{aligned}$$

We can also define the outlet resistance as:

$$\begin{aligned} R_{out}=kR_{ch}\#\left( S8 \right) \end{aligned}$$

where, $k$ is a proportionality constant based on the device geometry. For an idealized device with the same channel cross-section throughout, the length of the outlet channel would be $k$ times the length of the channel between each well. In an actual device, it is prudent to design an outlet channel that quickly widens to minimize the outlet resistance, so the value of $k$ would need to be calculated or derived from simulations. This allows us to rewrite Equation (S1) as:

$$\begin{aligned} P_{n}=P_{out}+\left( N-n+k \right)QR_{ch}\#\left( S9 \right) \end{aligned}$$

We can substitute Equations (S2-4), and (S7) into Equation (S9) to obtain:

$$\begin{aligned} \hat{p}_{n}P_{max}=\hat{p}_{out}P_{max}+\left( N-n+k \right)\hat{q}\left( \frac{2P_{max}}{\left( N-1 \right)R_{ch}} \right)R_{ch}\#\left( S10 \right) \end{aligned}$$

And simplify to:

$$\begin{aligned} \hat{p}_{n}=\hat{p}_{out}+2\hat{q}\left( \frac{N-n+k}{N-1} \right)\#\left( S11 \right) \end{aligned}$$

It is also possible to isolate the influence of well number by scaling $n$ and $k$ by the number of wells:

$$\begin{aligned} \hat{n}=\frac{n}{N}, \hat{k}=\frac{k}{N}\#\left( S12,S13 \right) \end{aligned}$$

This results in a final equation for the dimensionless pressure:

$$\begin{aligned} \hat{p}_{\hat{n}}=\hat{p}_{out}+2\hat{q}\left( 1-\hat{n}+\hat{k} \right)\left( \frac{1}{1-1/N} \right)\#\left( S14 \right) \end{aligned}$$

where $\hat{n}$ ranges from $1/N$ (first well) to 1 (last well) and $\hat{k}$ ranges from 0 (no outlet resistance) to infinity (infinite outlet resistance). This dimensionless equation makes it clear that while the effect of the radius and surface tension are removable, the effect of well number ($N$) is not entirely removable. However, the difference diminishes with increasing $N$. For the devices fabricated here, it scales the pressure drop across the wells by ~1.07. For very large $N$:

$$\begin{aligned} \hat{p}_{n}\approx\hat{p}_{out}+2\hat{q}\left( 1-\hat{n}+\hat{k} \right)\#\left( S15 \right) \end{aligned}$$

and $\hat{n}$ ranges from 0 to 1.

For any $\hat{q}\in[0,1)$, there are a range of possible outlet pressures. The lowest outlet pressure occurs when the last well is at the minimum possible pressure ($\hat{p}_{\hat{n}}=\hat{p}_{1}=-1$):

$$\begin{aligned} \hat{p}_{1}=-1=\hat{p}_{out}+2\hat{q}\left( 1-1+\hat{k} \right)\left( \frac{1}{1-1/N} \right)\to\hat{p}_{out}^{min}=-1-2\hat{q}\left( \frac{\hat{k}}{1-1/N} \right)\#\left( S16 \right) \end{aligned}$$

The highest outlet pressure occurs when the first well is at maximum pressure ($\hat{p}_{\hat{n}}=\hat{p}_{1/N}=1$):

$$\begin{aligned} \hat{p}_{1/N}=1=\hat{p}_{out}+2\hat{q}\left( 1-1/N+\hat{k} \right)\left( \frac{1}{1-1/N} \right)\to\hat{p}_{out}^{max}=1-2\hat{q}\left( \frac{1-1/N+\hat{k}}{1-1/N} \right)\#\left( S17 \right) \end{aligned}$$

For very large $N$, Equations (S16) and (S17) simplify to:

$$\begin{aligned} -1-2\hat{q}\hat{k}\lesssim\hat{p}_{out}\lesssim1-2\hat{q}\left( 1+\hat{k} \right)\#\left( S18 \right) \end{aligned}$$

For $q=0$, no flow occurs so the entire system pressure is set by the outlet. Equation (S18) then simplifies such that $-1<\hat{p}_{out}<1$.

When $q=1$, the first well will be at the maximum possible pressure ($\hat{p}_{\hat{n}}=\hat{p}_{1/N}=1$) and the last well will be at the minimum possible pressure ($\hat{p}_{\hat{n}}=\hat{p}_{1}=-1$). This means that the only possible outlet pressure is:

$$\begin{aligned} \hat{p}_{out}^{min}=-1-2\left( \frac{\hat{k}}{1-1/N} \right)=\hat{p}_{out}^{max}=1-2\left( \frac{1-1/N+\hat{k}}{1-1/N} \right)\#\left( S19 \right) \end{aligned}$$

These equations are demonstrated in Figure S1.1. It can be observed that at $\hat{q}=0$, the outlet pressure ranges between 0 and 1. As $\hat{q}$ increases, the available pressure range becomes narrower until only a single value is possible at $\hat{q}=1$.


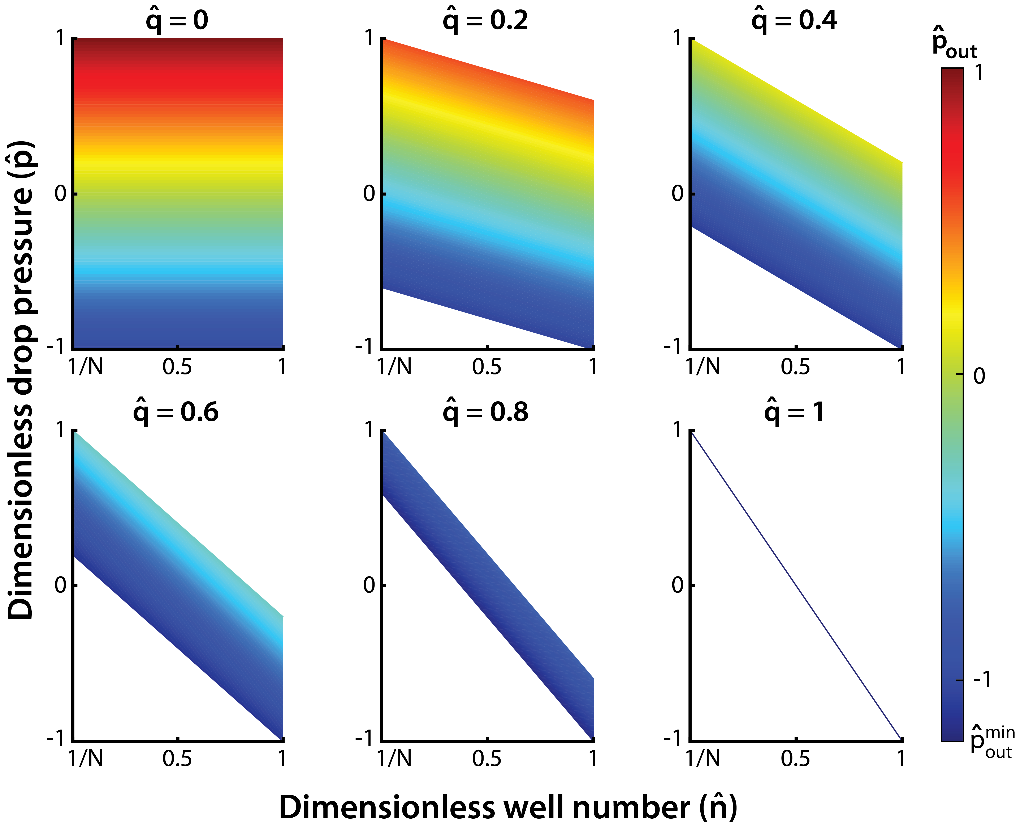


**Figure S1.1** Dimensionless drop pressures as a function of well number and outlet pressure under the full range of flow rates. The outlet pressure range narrows as the flow rate increases. (N=1000, k=100)

The volume can be similarly nondimensionalized. By setting:

$$\begin{aligned} \hat{v}=\frac{V}{V_{max}}\#\left( S20 \right) \end{aligned}$$

with:

$$\begin{aligned} V_{max}=\frac{2\pi r_{w}^{3}}{3}\#\left( S21 \right) \end{aligned}$$

for a hemispherical drop at maximum volume. These can be substituted in Equation (16) from the main text:

$$\begin{aligned} \hat{v}\left( \hat{p} \right)=\left\{ \begin{aligned} \frac{\pi{r_{w}}^{3}}{3\hat{p}^{3}}\left( 2-\left( 2+\hat{p}^{2} \right)\sqrt{1-\hat{p}^{2}} \right)/V_{max}, &0<\left| \hat{p} \right|\leq1 \\ 0/V_{max}, &\hat{p}=0 \end{aligned} \right.\#\left( S22 \right) \end{aligned}$$

and simplified to:

$$\begin{aligned} \hat{v}\left( \hat{p} \right)=\left\{ \begin{aligned} \frac{1}{2\hat{p}^{3}}\left( 2-\left( 2+\hat{p}^{2} \right)\sqrt{1-\hat{p}^{2}} \right), &0<\left| \hat{p} \right|\leq1 \\ 0, &\hat{p}=0 \end{aligned} \right.\#\left( S23 \right) \end{aligned}$$

Using the same values from Figure S1.1, we can plot the dimensionless volume in Figure S1.2. The same trend is visible, but with the nonlinearity introduced by the pressure-volume relationship.


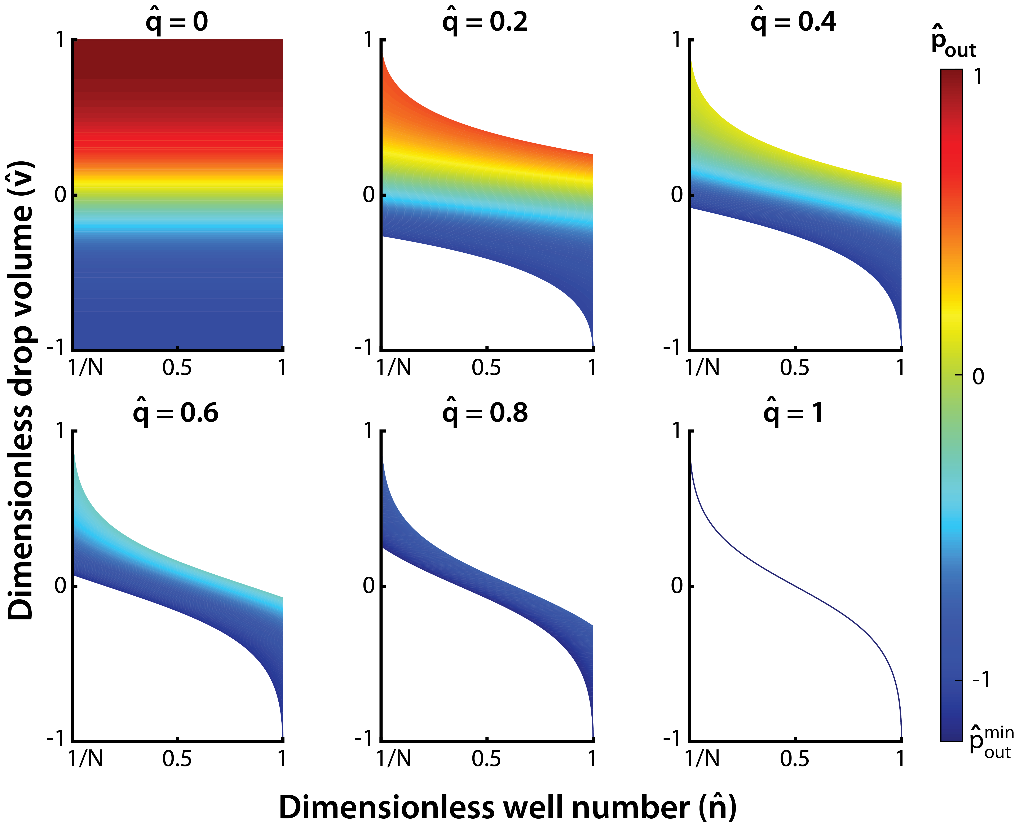


**Figure S1.2.** Dimensionless drop volumes as a function of well number and outlet pressure under the full range of flow rates. The outlet pressure range narrows as the flow rate increases. (N=1000, k=100)

Assuming a large well number, Figure S1.2 encompasses the full operating range for this type of device with a certain geometry characterized by $\hat{k}$. Changes to the radius or surface tension simply scale the pressure units, provided the radius is still small enough to be covered by the low-Bond number assumption described in the main text.

The factors that have a major effect on the fundamental operation of the device are the outlet pressure, flow rate, and the geometric parameter $\hat{k}$, which is related to the device geometry defining the channel and outlet resistances. The number of wells has only a minor effect near the well number of our fabricated device. Figure S1.3 demonstrates the effect of changing $k$ from 100 to 1000, effectively increasing the outlet resistance by ten times. While the shape of the plot is similar, $\hat{p}_{out}^{min}$ is substantially reduced. The higher outlet resistance requires that the outlet pressure be brought much lower to create the same pressure difference and drive the same flow rate. This can lead to bubble expansion and clogging, as discussed in the main text.


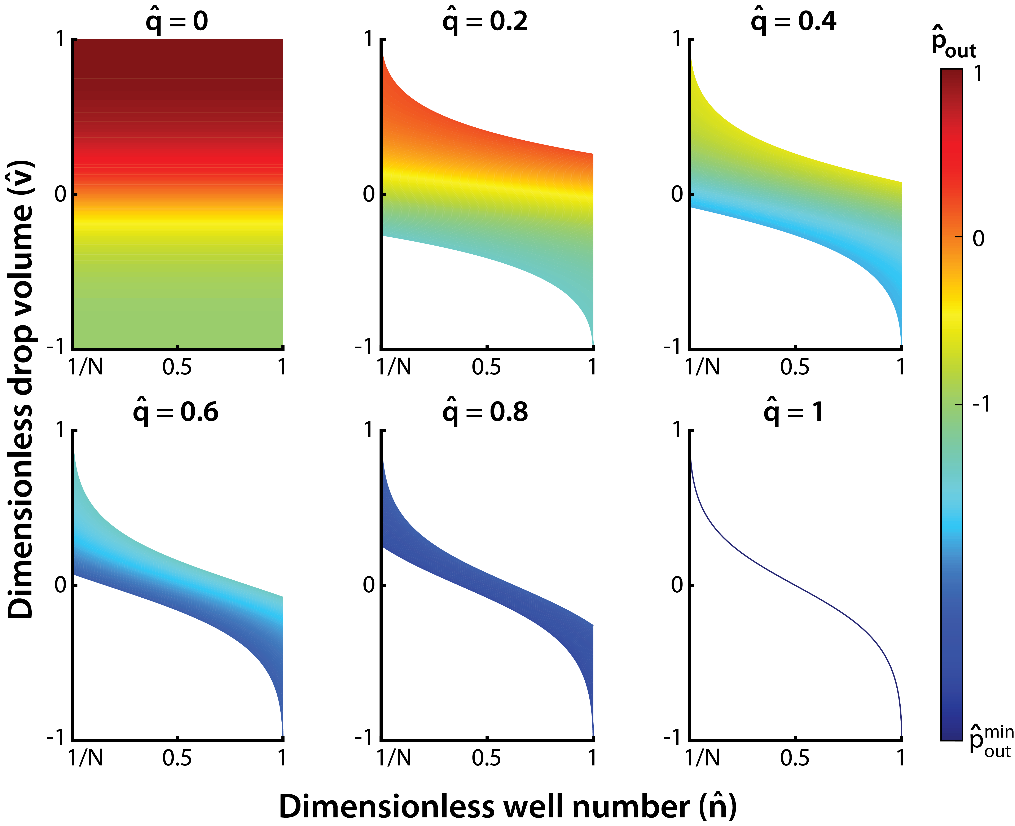


**Figure S1.3.** Dimensionless drop volumes as a function of well number and outlet pressure under the full range of flow rates. The outlet pressure range narrows as the flow rate increases. (N=1000, k=1000)

**Note 2**

**Derivation of volume-pressure relationship for a hanging drop fluidic capacitor**

Starting with the typical equation for a positive-volume spherical cap in terms of $h$ and $r$:

$$\begin{aligned} V=\frac{\pi}{3}h^{2}\left( 3r-h \right)\#\left( S24 \right) \end{aligned}$$

We can substitute $h=r-\sqrt{r^{2}-r_{w}^{2}}$, where $r_{w}$ refers to the radius of the well, to obtain:

$$\begin{aligned} V=\frac{\pi}{3}\left( 2r^{3}-\left( 2r^{2}+{r_{w}}^{2} \right)\sqrt{r^{2}-{r_{w}}^{2}} \right)\#\left( S25 \right) \end{aligned}$$

which must be expressed as a piecewise function to encompass both concave and convex drops:

$$\begin{aligned} V\left( r \right)=\left\{ \begin{aligned} \frac{\pi}{3}\left( 2r^{3}-\left( 2r^{2}+{r_{w}}^{2} \right)\sqrt{r^{2}-{r_{w}}^{2}} \right), &r_{w}\leq r<\infty\\ -\frac{\pi}{3}\left( 2\left| r \right|^{3}-\left( 2r^{2}+{r_{w}}^{2} \right)\sqrt{r^{2}-{r_{w}}^{2}} \right), &-r_{w}\geq-r>-\infty\end{aligned} \right.\#\left( S26 \right) \end{aligned}$$

as discussed in the main text. This is undefined for $-r_{w}<r<r_{w}$. We can express this in terms of pressure by substituting a rearranged Young-Laplace equation:

$$\begin{aligned} r=\frac{2\sigma}{P}\#\left( S27 \right) \end{aligned}$$

We will reduce $\Delta P$ to $P$ for simplicity, as all pressures in the system are considered in reference to the same external atmospheric pressure. This results in:

$$\begin{aligned} V\left( P \right)=\frac{\pi}{3P^{3}}\left( 16\sigma^{3}-\left( 8\sigma^{2}+{r_{w}}^{2}P^{2} \right)\sqrt{4\sigma^{2}-{r_{w}}^{2}P^{2}} \right)\#\left( S28 \right) \end{aligned}$$

Note that because the $P^{-3}$ term results in a sign difference for positive and negative pressures, this equation is valid for both convex and concave drops. However, it has a discontinuity at $P=0$. Evaluating the limit as $P$ approaches 0 results in a $\left( \infty\right)\left( 0 \right)$ indeterminate form. By rearranging to:

$$\begin{aligned} V\left( P \right)=\frac{\left( 16\sigma^{3}-\left( 8\sigma^{2}+{r_{w}}^{2}P^{2} \right)\sqrt{4\sigma^{2}-{r_{w}}^{2}P^{2}} \right)}{\frac{3P^{3}}{\pi}}\#\left( S29 \right) \end{aligned}$$

we obtain a 0/0 indeterminate form and can apply L’Hôpital’s rule:

$$\begin{aligned} \lim_{P\to0} \frac{\frac{d}{dP}\left( 16\sigma^{3}-\left( 8\sigma^{2}+{r_{w}}^{2}P^{2} \right)\sqrt{4\sigma^{2}-{r_{w}}^{2}P^{2}} \right)}{\frac{d}{dP}\frac{3P^{3}}{\pi}}\left( 0/0\text{indeterminate} \right)\#\left( S30 \right) \end{aligned}$$

$$\begin{aligned} =\lim_{P\to0}\frac{3{r_{w}}^{4}P^{3}\left( 4\sigma^{2}-{r_{w}}^{2}P^{2} \right)^{-\frac{1}{2}}}{\frac{9P^{2}}{\pi}}=\lim_{P\to0}\frac{\pi{r_{w}}^{4}P}{3\sqrt{4\sigma^{2}-{r_{w}}^{2}P^{2}}}=0 \end{aligned}$$

Therefore, the discontinuity is removable, and we can define:

$$\begin{aligned} V\left( P \right)=\left\{ \begin{aligned} \frac{\pi}{3P^{3}}\left( 16\sigma^{3}-\left( 8\sigma^{2}+{r_{w}}^{2}P^{2} \right)\sqrt{4\sigma^{2}-{r_{w}}^{2}P^{2}} \right), &0<\left| P \right|<\frac{2\sigma}{r_{w}} \\ 0, &P=0 \end{aligned} \right.\#\left( S31 \right) \end{aligned}$$

To obtain a constitutive equation analogous to the capacitor equation:

$$\begin{aligned} i=\frac{dq}{dt}=C\frac{dv}{dt} \text{with }C=\frac{dq}{dv}\#\left( S32 \right) \end{aligned}$$

we can replace each term with its fluid analog, using:

$$i \left( \mathrm{current}\left[ A \right] \right)=Q \left( volumetric flow rate \left[ {m^{3}}/s \right] \right)$$

$$q \left( charge [C] \right)=V (volume [m^{3}])$$

$$v \left( voltage [V] \right)=\Delta P (pressure [Pa])$$

$$C \left( capacitance [F] \right)=C (compliance [{m^{3}}/\text{Pa}])$$

The hanging drop volume-pressure relationship cannot be reasonably approximated as linear for extreme pressure values. Therefore, the compliance cannot be considered as a single value. Instead, we must calculate:

$$\begin{aligned} \frac{dV}{dP}=\frac{dV}{dr}\frac{dr}{dP}=\frac{d}{dr}\left( \frac{\pi}{3}\left( 2r^{3}-\left( 2r^{2}+{r_{w}}^{2} \right)\sqrt{r^{2}-{r_{w}}^{2}} \right) \right)\cdot\frac{d}{dP}\left( \frac{2\sigma}{P} \right)\#\left( S33 \right) \end{aligned}$$

$$=\pi r\left( 2r+\frac{{r_{w}}^{2}-2r^{2}}{\sqrt{r^{2}-{r_{w}}^{2}}} \right)\left( -\frac{2\sigma}{P^{2}} \right)$$

Further substituting the Young-Laplace equation results in:

$$V\left( P \right)$$

$$\begin{aligned} V^{'}\left( P \right)=\frac{4\pi\sigma^{2}}{P^{4}}\left( \frac{8\sigma^{2}-{r_{w}}^{2}P^{2}}{\sqrt{{4\sigma}^{2}-{r_{w}}^{2}P^{2}}}-4\sigma\right)\#\left( S34 \right) \end{aligned}$$

This can be evaluated for all pressures within $(-P_{max},P_{max})$ except at $P=0$. We can follow the same process as for $V(P)$ and take the limit as $P$ approaches zero.

$$\begin{aligned} \lim_{P\to0} \frac{\frac{8\sigma^{2}-{r_{w}}^{2}P^{2}}{\sqrt{{4\sigma}^{2}-{r_{w}}^{2}P^{2}}}-4\sigma}{\frac{P^{4}}{4\pi\sigma^{2}}}\left( 0/0\text{indeterminate} \right)\#\left( S35 \right) \end{aligned}$$

Applying L’Hôpital’s rule results in:

$$\begin{aligned} \lim_{P\to0} \frac{\frac{d}{dP}\left( \frac{8\sigma^{2}-{r_{w}}^{2}P^{2}}{\sqrt{{4\sigma}^{2}-{r_{w}}^{2}P^{2}}}-4\sigma\right)}{\frac{d}{dP}\frac{P^{4}}{4\pi\sigma^{2}}}=\lim_{P\to0} \frac{{r_{w}}^{4}P^{3}\left( 4\sigma^{2}-{r_{w}}^{2}P^{2} \right)^{-\frac{3}{2}}}{\frac{P^{3}}{\pi\sigma^{2}}}=\lim_{P\to0} \frac{\pi{r_{w}}^{4}\sigma^{2}}{\left( 4\sigma^{2}-{r_{w}}^{2}P^{2} \right)^{\frac{3}{2}}}=\frac{\pi{r_{w}}^{4}}{8\sigma}\#\left( S36 \right) \end{aligned}$$

Therefore, this is also a removable discontinuity and we can define:

$$\begin{aligned} V^{'}\left( P \right)=\left\{ \begin{aligned} \frac{4\pi\sigma^{2}}{P^{4}}\left( \frac{8\sigma^{2}-{r_{w}}^{2}P^{2}}{\sqrt{{4\sigma}^{2}-{r_{w}}^{2}P^{2}}}-4\sigma\right), &0<\left| P \right|<\frac{2\sigma}{r_{w}} \\ \frac{\pi{r_{w}}^{4}}{8\sigma}, &P=0 \end{aligned} \right.\#\left( S37 \right) \end{aligned}$$

Using this equation, we can express the constitutive equation for the fluidic capacitor as:

$$Q=\frac{dV}{dt}=C\frac{dP}{dt} \text{with} C=V^{'}\left( P \right) \text{as defined in eq. S37}$$

This allows us to define differential equations to describe multiple drop systems.

These equations can be further simplified by introducing a parameter $\hat{p}=\frac{P}{P_{\max}}$, where $P_{max}=\frac{2\sigma}{r_{w}}$ is the maximum pressure difference for the drop. This results in $P=\frac{2\sigma\hat{p}}{r_{w}}$ and ${r_{w}}^{2}P^{2}=4\sigma^{2}\hat{p}^{2}$. Equations (S31) and S37 can then be simplified to:

$$\begin{aligned} V\left( \hat{p} \right)=\left\{ \begin{aligned} \frac{\pi{r_{w}}^{3}}{3\hat{p}^{3}}\left( 2-\left( 2+\hat{p}^{2} \right)\sqrt{1-\hat{p}^{2}} \right), &0<\left| \hat{p} \right|\leq1 \\ 0, &\hat{p}=0 \end{aligned} \right.\#\left( S38 \right) \end{aligned}$$

$$\begin{aligned} V^{'}\left( \hat{p} \right)=\left\{ \begin{aligned} \frac{\pi{r_{w}}^{4}}{2\sigma\hat{p}^{4}}\left( \frac{2-\hat{p}^{2}}{\sqrt{1-\hat{p}^{2}}}-2 \right), &0<\left| \hat{p} \right|<1 \\ \frac{\pi R^{4}}{8\sigma}, &\hat{p}=0 \end{aligned} \right.\#\left( S39 \right) \end{aligned}$$

**Note 3**

**Numerical solution for multiple drop systems**

By summing the flow at each node, we can define the pressures and flows for each well as:

$$\begin{aligned} \begin{matrix} P_{1}\left( t \right)=P_{in}\left( t \right)-q_{in}\left( t \right)R_{in} \\ P_{2}\left( t \right)=P_{1}\left( t \right)-q_{1}\left( t \right)R_{1} \\ \begin{matrix} \vdots\\ P_{out}\left( t \right)=P_{n}\left( t \right)-q_{out}\left( t \right)R_{out} \end{matrix} \end{matrix}\text{with }\begin{matrix} q_{in}\left( t \right)=q_{C1}\left( t \right)+q_{1}\left( t \right) \\ q_{1}\left( t \right)=q_{C2}\left( t \right)+q_{2}\left( t \right) \\ \begin{matrix} \vdots\\ q_{4}\left( t \right)=q_{C5}\left( t \right)+q_{out}\left( t \right) \end{matrix} \end{matrix}\#(S40) \end{aligned}$$

where $q_{Cn}$ denotes the flow into the nth well corresponding to a volume change of the nth drop. We can rearrange these to the general form:

$$\begin{aligned} P_{n}\left( t \right)=P_{n-1}\left( t \right)-q_{n-1}\left( t \right)R_{n-1}=P_{n-1}\left( t \right)-(q_{Cn}\left( t \right)+q_{n}\left( t \right))R_{n-1}\#\left( S41 \right) \end{aligned}$$

By equating:

$$\begin{aligned} q_{Cn}=\frac{dV_{Cn}}{dt}=\frac{dV_{Cn}}{dP_{n}}\frac{dP_{n}}{dt}\#\left( S42 \right) \end{aligned}$$

we can write:

$$\begin{aligned} P_{n}\left( t \right)=P_{n-1}\left( t \right)-q_{n}\left( t \right)R_{n-1}-\frac{dV_{Cn}}{dP_{n}}\frac{dP_{n}}{dt}R_{n-1}=P_{n-1}\left( t \right)-q_{n}\left( t \right)R_{n-1}-V^{'}\left( P_{n}\left( t \right) \right)P_{n}^{'}\left( t \right)R_{n-1}\#\left( S43 \right) \end{aligned}$$

and rearrange to:

$$\begin{aligned} P_{n}^{'}\left( t \right)=\frac{P_{n-1}\left( t \right)-q_{n}\left( t \right)R_{n-1}-P_{n}\left( t \right)}{V^{'}\left( P_{n}\left( t \right) \right)R_{n-1}}\#\left( S44 \right) \end{aligned}$$

with:

$$\begin{aligned} q_{n}\left( t \right)=\frac{P_{n}\left( t \right)-P_{n+1}\left( t \right)}{R_{n}} \#\left( S45 \right) \end{aligned}$$

With $P_{n}^{'}\left( t \right)$ entirely in terms of pressures and resistances, it is possible to solve with a typical ordinary differential equation solver. When solving differential equations for $V^{'}\left( P \right)$ numerically, a major challenge is the numerical instability introduced as $P$ approaches zero because the product tends towards $\left( \infty\right)\left( 0 \right)$. To mitigate this, we can evaluate $V^{'}\left( P \right)$ using the value at the removable discontinuity within a window near $P=0$ rather than at the exact value:

$$\begin{aligned} V^{'}\left( P \right)=\left\{ \begin{aligned} \frac{4\pi\sigma^{2}}{P^{4}}\left( \frac{8\sigma^{2}-{r_{w}}^{2}P^{2}}{\sqrt{{4\sigma}^{2}-{r_{w}}^{2}P^{2}}}-4\sigma\right), &P_{w}<\left| P \right|<\frac{2\sigma}{r_{w}} (\text{case a}) \\ \frac{\pi{r_{w}}^{4}}{8\sigma}, &|P|\leq P_{w} (\text{case b}) \end{aligned} \right.\#\left( S46 \right) \end{aligned}$$

The window should be chosen to exclude any large instability near zero, which will depend on the specifics of the system used. However, it is also important to evaluate the error that this approximation introduces. For a given pressure, $P_{w}$, because the result of case a is always greater than the result of case b, we can evaluate the error as:

$$\begin{aligned} \epsilon\left( P_{w} \right)=1-\frac{\frac{\pi{r_{w}}^{4}}{8\sigma}}{\frac{4\pi\sigma^{2}}{P_{w}^{4}}\left( \frac{8\sigma^{2}-{r_{w}}^{2}P_{w}^{2}}{\sqrt{{4\sigma}^{2}-{r_{w}}^{2}P_{w}^{2}}}-4\sigma\right)}\#\left( S47 \right) \end{aligned}$$

To simplify, we can introduce a dimensionless parameter $\alpha$ such that:

$$\begin{aligned} P_{w}=\alpha P_{max}=\alpha\frac{2\sigma}{r_{w}}\to P_{w}r_{w}=2\sigma\alpha\#\left( S48 \right) \end{aligned}$$

Substituting and simplifying results in:

$$\begin{aligned} \epsilon\left( \alpha\right)=1-\frac{\alpha^{4}\sqrt{1-\alpha^{2}}}{4\left( 2-\alpha^{2}-2\sqrt{1-\alpha^{2}} \right)}\#\left( S49 \right) \end{aligned}$$

This equation allows for reasonable selection of a window. In MATLAB R2022a (win64), for example, the following code:

pressure = -950:0.001:950; %Pressure [Pa]

radius = 150e-6; %Well radius [m]

tension = 0.072; %Surface tension (Water at room temperature) [N/m]

dVdP = @(P,R,T) (4*pi*T^2./P.^4).*((8*T^2-R^2.*P.^2)./sqrt(4*T^2-R^2.*P.^2)-4*T);

plot(pressure,dVdP(pressure,radius,tension)*10^12) %convert from m^3 to nL

yline(pi*(radius)^4/(8*tension)*10^12) %plot value at discontinuity (S3.b)

results in the large oscillations near $P=0$ visible in Figure S3.1.

**
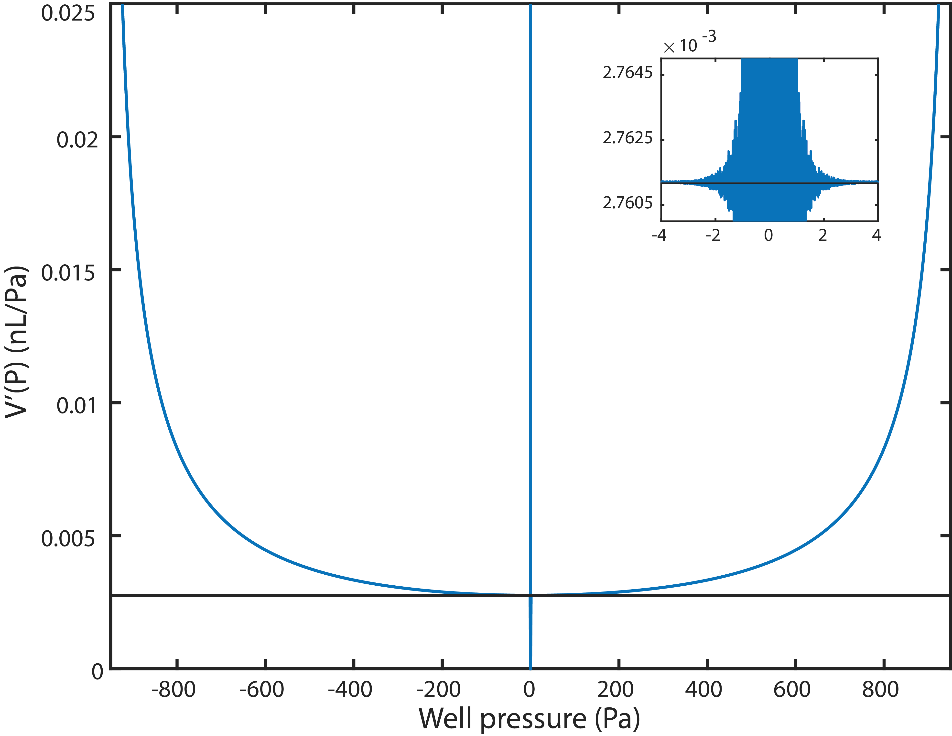
**

**Fig S3.1:** Instability is observed when calculating $V^{'}\left( P \right)$ for small pressure values (in this case, less than about 4 Pa). The horizontal black line shows the value of $V^{'}\left( P \right)$ at the discontinuity.

For the devices considered in the main text, $P_{w}$ was chosen as 4 Pa while $P_{max}$ was 960 Pa. This results in $\alpha=0.004$ and a maximum error of approximately 0.001% when evaluating $V^{'}\left( P \right)$. Even for a value $\alpha=0.1$, the maximum error remains at approximately 1% because $V^{'}\left( P \right)$ is nearly horizontal throughout this range.

The full code used to solve multiple drop systems in MATLAB is given here:

function Well_diffeq_solver_example()

% Main function to run the solver for multiple in-series wells

% Matthew Wester (LIBNA, UIUC, mwester3@illinois.edu)

% May 2024

%% Initial conditions/parameters

%Constants

mu = 8.90e-4; %dynamic viscosity (water, room temp) [Pa.s]

T = 0.072; %surface tension (water, room temp) [N/m]

%Device characteristics

num_wells = 5; %number of series wells

Rad = 150e-6; %well radius [m]

%Channel dimensions

%between wells

width_ch = 100*10^-6; height_ch = 10*10^-6; length_ch = 20*10^-6; %Channel dimensions [m]

%at inlet/outlet

width_ch1 = 420*10^-6; height_ch1 = 10*10^-6; length_ch1 = 4*10^-3;

width_ch2 = 100*10^-6; height_ch2 = 10*10^-6; length_ch2 = 500*10^-6; %m

%Resistor calculations (inlet/outlet have two channel sizes)

%for rectangular channel, R~(12*mu*L)/((w*h^3)*(1-0.63*(h/w))) for L>>w, w>>h

R_ch_calc = (12*mu*length_ch)/((width_ch*height_ch^3)*(1-0.63*height_ch/width_ch));

R_ch1 = (12*mu*length_ch1)/((width_ch1*height_ch1^3)*(1-0.63*height_ch1/width_ch1));

R_ch2 = (12*mu*length_ch2)/((width_ch2*height_ch2^3)*(1-0.63*height_ch2/width_ch2));

adj = [0.6 1]; %proportional adjustment to match experimental observation (default 1)

R_port_calc = R_ch1+R_ch2; R_ch = adj(1)*R_ch_calc; R_o = adj(2)*R_port_calc;

Res = [R_o R_ch*ones(1,num_wells-1) R_o]';

%Inlet/Outlet functions

%example - sine wave, outlet at constant

syms x P_in_ex(x) P_out_ex(x)

f = 20; %Hz

offset = 2000; %Pa

amp = 500; %Pa

%P_init = zeros(num_wells,1); %initial well pressures [Pa], optional

P_in_ex(x) = offset+amp*sin(2*pi*f*x);

P_out_ex(x) = -500;

t_bounds = [0 10]; %observation time [s]

t_out = 1/(20*f); %output period for plotting [s]

[t_ex,P_ex] = series_solver({P_in_ex,P_out_ex,x},Res,T,Rad,t_bounds,t_out); %solve

%% Plot

t_plot = [10-3/f 10]; %time range to plot [start stop]

[ax1,ax2] = plot_pressures(t_ex,P_ex,t_plot,t_out);

linkaxes([ax1 ax2],'x')

end

function [ax1,ax2] = plot_pressures(t,P,t_plot,t_out)

%Plots outcome of single system solution

%Inputs: t - nx1 array of time points for solution [s]

% P - nx(num_wells+2) pressures at inlet, each well, and outlet for solution [Pa]

% t_plot - [start stop] time range to plot [s]

% t_out - period of time data [s]

%Outputs: ax1 - axes for left subplot (pressure difference)

% ax2 - axes for right subplot (well pressures)

figure;

indexes = round(t_plot*(1/t_out)+1); %select data for specified plotted time

if indexes(2) > length(t)

indexes(2) = length(t);

end

subplot(1,3,1); %plot pressure difference (in-out)

Pdiff1 = P(:,1)-P(:,end);

plot(t(indexes(1):indexes(2))-t(indexes(1)),Pdiff1(indexes(1):indexes(2)));

title('P_{in}-P_{out} (Pa)');

xlabel('time (s)');

ax1 = gca;

subplot(1,3,2:3); %plot well pressures

plot(t(indexes(1):indexes(2))-t(indexes(1)),P(indexes(1):indexes(2),2:end-1),'-');

title('Well pressure (Pa)');

xlabel('time (s)');

ax2 = gca;

leg = legend(arrayfun(@num2str,1:(size(P,2)-2), 'UniformOutput', 0),'AutoUpdate','off','Location','eastoutside');

title(leg,'Well');

end

function [t,P] = series_solver(in_out,Res,T,Rad,t_bounds,t_out,P_init)

%Solves differential equations using built-in ode solver to determine well pressures

%Inputs: in_out - a 1x3 array [P_in P_out x] containing:

% P_in - a continuously differentiable symbolic function of the inlet pressure [Pa] in terms of a single variable x [s]

% P_out - a continuously differentiable symbolic function of the outlet pressure [Pa] in terms of a single variable x [s]

% x - a symbolic variable [s]

% Res - (num_wells+1)x1 array of resistances for the inlet, each channel, and outlet [Pa/(m^3/s)]

% T - surface tension [N/m]

% Rad - drop base radius [m]

% t_bounds - [start_time end_time] to run the ode solver over [s]

% t_out - output period desired for solution [s]

% P_init (optional) - (num_wells)x1 array of initial well pressures [Pa]

%Outputs: t - nx1 array of time points for solution [s]

% P - nx(num_wells+2) pressures at inlet, each well, and outlet for solution [Pa]

P_in = in_out{1};

P_out = in_out{2};

x = in_out{3};

if nargin > 6

init = [P_in(t_bounds(1)); P_init; P_out(t_bounds(1))]; %set to initial pressures given

else

init = [P_in(t_bounds(1)); steady_solver(P_in(t_bounds(1)),P_out(t_bounds(1)),Res); P_out(t_bounds(1))]; %approximate initial pressures assuming steady state

end

t_span = t_bounds(1):t_out:t_bounds(2); %determine time points to force ode output

dP_in = matlabFunction(diff(P_in,x));

dP_out = matlabFunction(diff(P_out,x));

[t,P] = ode89(@(t,P) pressure(t,P,dP_in,dP_out,Res,T,Rad),t_span,double(init)); %run ode solver

end

function Pressures = steady_solver(P_in,P_out,Res)

%Basic approximation of well pressures at steady state

%Inputs: P_in - inlet pressure [Pa]

% P_out - outlet pressure [Pa]

% Res - (num_wells+1)x1 array of resistances for the inlet, each channel, and outlet [Pa/(m^3/s)]

%Oututs: Pressures - 1xnum_wells array with steady-state pressures of each well

Q = (P_in-P_out)/sum(Res); %calculate SS flow rate

P_drops = Q*Res(2:end); %calculate pressure drop between each node

Pressures = cumsum(P_drops,'reverse')+P_out;

end

function dPdt = pressure(t,pressures,dP_in,dP_out,Res,T,Rad)

%Calculates dP/dt for a sherical drop at a given instant

%Inputs: t - time point [s]

% pressures - (num_wells+2)x1 array of pressures at the inlet, each well, and outlet [Pa]

% dP_in - a symbolic function, the derivative of the inlet pressure [Pa] in terms of a single variable t [s]

% dP_out - a symbolic function, the derivative of the outlet pressure [Pa] in terms of a single variable t [s]

% Res - (num_wells+1)x1 array of resistances for the inlet, each channel, and outlet [Pa/(m^3/s)]

% T - surface tension [N/m]

% Rad - drop base radius [m]

%Outputs: dPdt - (num_wells+2)x1 array containing the dP/dt value for the inlet, each well, and outlet at time t [Pa]

num_wells = length(Res)-1; %find number of wells

dPdt = zeros(num_wells+2,1); %initialize zero dP array

dPdt(1,1) = dP_in(t); %set inlet dP

qs = zeros(num_wells+1,1); %initialize zero array for flows between nodes

qs(1) = (pressures(1,1)-pressures(2,1))/Res(1,1); %calculate inlet flow

qcs = zeros(num_wells,1); %initialize zero array for flow into wells

dVdPs = zeros(num_wells+1,1); %initialize zero dV/dP array

for n = 2:num_wells+1 %iterate over remaining nodes

qs(n) = (pressures(n,1)-pressures(n+1,1))/Res(n,1); %calculate flow out of node

dVdPs(n-1) = dVdP_piecewise(T,Rad,pressures(n,1)); %calculate well dV/dP at current pressure

qcs(n-1) = (pressures(n,1)-pressures(n-1,1)+qs(n)*Res(n-1,1))/(-Res(n-1,1)); %calculate flow into well

dPdt(n,1) = qcs(n-1)/dVdPs(n-1); %calculate dP/dt for well

end

dPdt(num_wells+2,1) = dP_out(t); %set outlet dP

end

function dVdP = dVdP_piecewise(T,Rad,P)

%Calculates dV/dP for a sherical drop based on the piecewise equation

% Valid from -2T/R<P<2T/R

%Inputs: T - surface tension [N/m]

% Rad - drop base radius [m]

% P - drop pressure (inside-outside) [Pa]

%Outputs: dVdP - dV/dP value [m^3/Pa]

P_w = 4; %value of pressure window, discussed in Supplementary Note 2

if abs(P) <= P_w %case b

dVdP = pi*Rad^4/(8*T);

elseif abs(P) < 2*T/Rad %case a

dVdP = (4*pi*T^2/P^4)*((8*T^2-Rad^2*P^2)/sqrt(4*T^2-Rad^2*P^2)-4*T);

else %outside of relevant range

dVdP = NaN;

end

end

**Note 4**

**Compliance of PTFE tubing**

Equation for compliance of cylindrical tubing (Eq. 1.73):^1^

$$\begin{aligned} C_{hyd}^{tube}=2\pi\left( 1+\bar{v} \right)\frac{\left( 1-2\bar{v} \right)+\left( 1+\delta\right)^{2}}{\left( 1+\delta\right)^{2}-1}\frac{a^{2}L}{Y} \text{with} \delta=\frac{d}{a}\#\left( S50 \right) \end{aligned}$$

where $\bar{v}$ is the Poisson ratio, $Y$ is Young’s modulus, $a$ is the tubing inner diameter, $L$ is the tubing length, and $d$ is the wall thickness.

For 1/16” OD (1/32” ID) PTFE tubing used commonly in microfluidics applications, $\bar{v}=0.45$, $Y=0.5 GPa$, $\delta=1$, and $a=397 \mu m$. As a result:

$$C_{hyd}^{tube}=\left( 3.92\times{10}^{-15} \right)L m^{3}/Pa$$

with $L$ in meters. For a tubing length of 10 cm, typical of the scale for flow experiments in this paper, the compliance is $3.92\times{10}^{-16} m^{3}/Pa$.

For a drop with a radius ($r$) of 150 µm, the minimum compliance is:

$$\begin{aligned} C_{min}=\frac{\pi{r_{w}}^{4}}{8\sigma}\#\left( S51 \right) \end{aligned}$$

with $\sigma=72 mN/m$ for water. The resulting compliance is $2.76\times{10}^{-15} m^{3}/Pa$, about ten times higher than the tubing for a single well. At $p=0.95$, where the drop is nearly full, the compliance is about a hundred times higher at $2.05\times{10}^{-14} m^{3}/Pa$.

1. Bruus, H. Chapter 1: Governing Equations in Microfluidics. In *Microscale Acoustofluidics* [Online]. (eds. Laurell, T., Lenshof; A.) 1-28 (Royal Society of Chemistry, 2014).

**Note 5**

**Fabrication challenges**

Two notable challenges were encountered in the fabrication of these devices. First, through-etching a wafer using DRIE to an oxide etch stop can result in notching defects. These occur when highly directional reactive ions are reflected by the oxide layer back into the feature side walls. The resulting damage is shown in Figure S4.1.


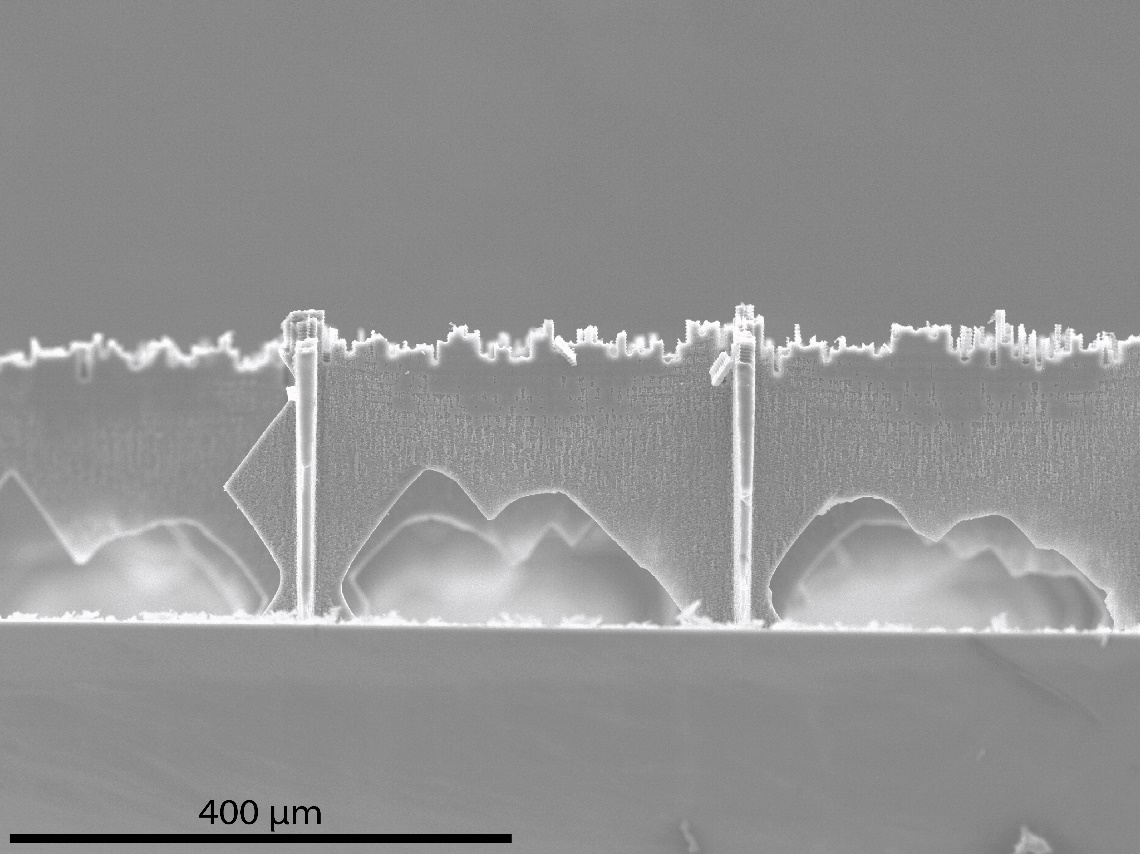


**Figure S5.1**. Notching effects at the bottom of the well can cause substantial damage to device features in less than a minute using high-etch-rate recipes. Also visible in this image is damage from photoresist breakdown on the device surface.

This has been mitigated in other devices that require through etching by completing this step before bonding.^1^ However, this was determined not to be feasible for our device because of the low contact area and fragile side walls formed during the well etching as well as the thickness of the glass. To mitigate this damage, a two-stage etch was developed. The high-frequency (13.56 MHz) platen of the DRIE tool was used to provide a high etch rate during most of the process. When nearing the buried oxide layer, the low-frequency (380 kHz) platen was utilized for a slower, gentler etch that would not cause notching defects as rapidly.

The second challenge was the fabrication of two features with drastically different depths (5 and 300 µm) on the same surface. There were two approaches that were possible in this situation: (1) etching the shallow features and then using a thick photoresist layer to form the second mask or (2) using a buried oxide mask and photoresist mask to pattern both layers before etching either. Both methods were tested - the fabrication schematics and results are shown in Figure S4.2.


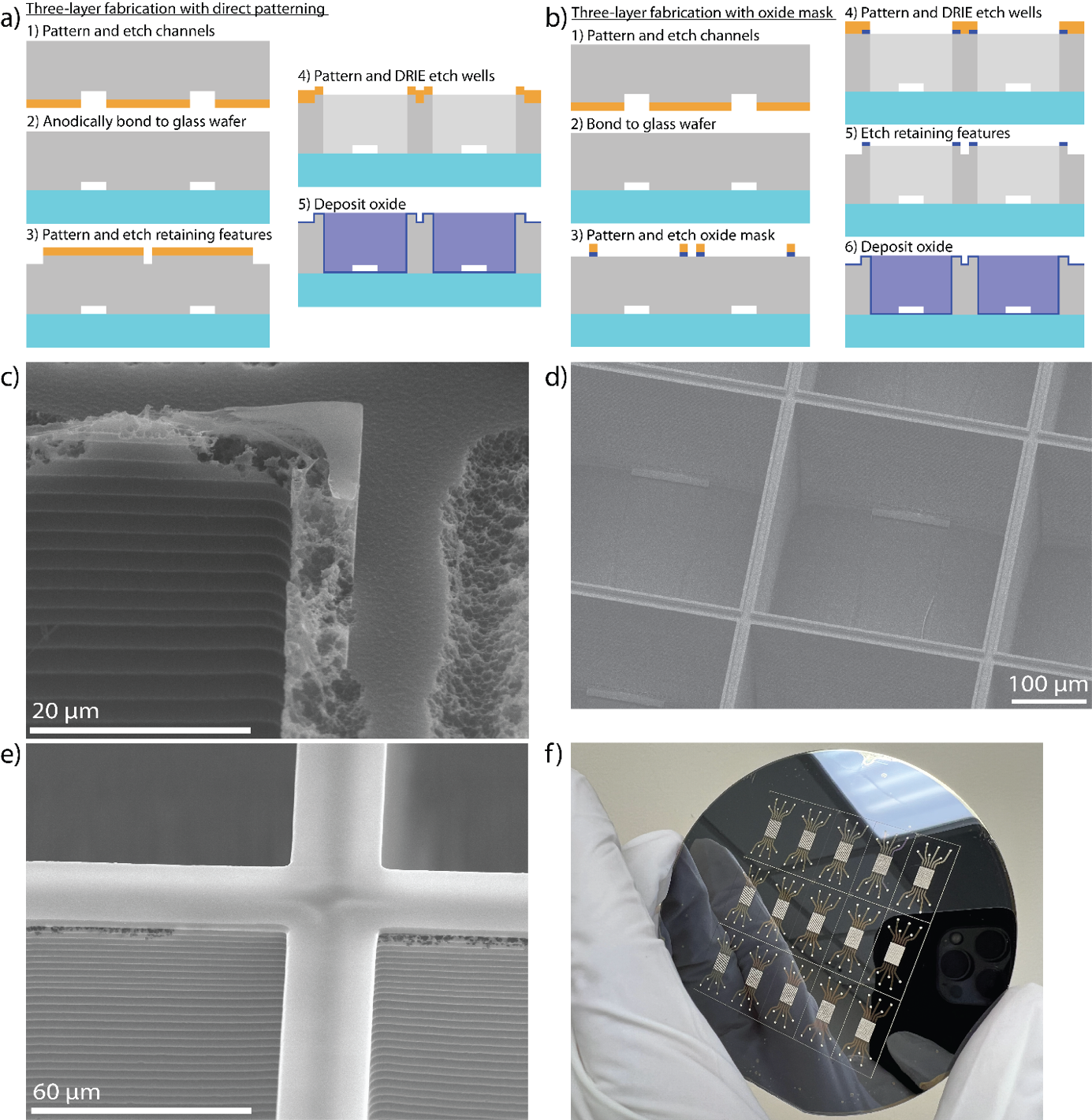


**Figure S5.2**. Two fabrication protocols (left column and right column) were tested to produce the deep wells and shallow surface features. Schematics for the two are presented in (a) and (b). (c) shows significant damage to the retaining features observed across multiple wafers while (d) shows intact, well-defined walls (e) Damage to the retaining features is apparent with photoresist still in place, indicating that damage occurs to the sidewalls during DRIE etching for reasons other than photoresist breakdown. (f) 15 devices were fabricated per 4” wafer.

Interestingly, although the photoresist integrity was maintained throughout the deep etch in the first option, significant damage to the well sidewalls occurred exclusively opposite the trench features. Upon removal of the photoresist, most of the retaining features were so damaged that they separated from the device entirely. This is possibly due to reduced thermal or charge dissipation from the retaining features when the retaining features are etched before the deep features. Ultimately, the second option was found to yield devices with the desired performance more consistently.

1. Frisk, T. W., Khorshidi, M. A., Guldevall, K., Vanherberghen, B. & Önfelt, B. A silicon-glass microwell platform for high-resolution imaging and high-content screening with single cell resolution. *Biomed Microdevices* **13**, 683–693 (2011).

**Experimental Details**

**Fabrication protocol**

All photomasks were designed in AutoCAD (Autodesk, San Francisco, CA, USA). Devices were fabricated on a 4”, 300-µm-thick DSP silicon wafer (Item #2345, University Wafer, Boston, MA, USA). A standard degreasing procedure of acetone, isopropanol, and water rinses followed by nitrogen drying was followed. Alignment marks were pre-patterned on each side of the wafer because the available direct write system was not equipped for back side alignment.

*Channel Layer Fabrication:* The silicon wafer was dehydrated for 2 minutes at 140˚C and spin coated with an approximately 5 µm layer of MEGAPOSIT SPR220-4.5 (Kayaku Advanced Materials, Westborough, MA, USA). It was soft baked at 60˚C for 2 min and 100˚C for 1 minute. The photoresist was patterned with the first mask layer (channels) using the MLA 150 Maskless Aligner (Heidelberg, Heidelberg, Germany) at 375 nm and a dose of approximately 300 mJ/cm^2^. It was developed in a 5:1 solution of DI water to AZ 400K developer (AZ Electronic Materials, Luxembourg) for approximately two minutes. After rinsing and drying, the wafer was hard baked for 3 minutes at 120˚C while covered. The channels were etched using the Bosch process in the STS Pegasus deep reactive ion etch (DRIE) tool (SPTS Technologies, Newport, UK), with a recipe optimized for creating extremely smooth but shallow features with vertical sidewalls; the recipe includes an O_2_ descum at the start. After confirming the correct etch depth with a stylus profilometer (Alpha-Step D-500, KLA Instruments, Milpitas, CA, USA), the remaining photoresist was stripped by submerging the wafer in MICROPOSIT Remover 1165 (Kayaku AM) heated to 80˚C for 10 min and then sonicated for 10 minutes at 60˚C. After another degrease, the wafer was dry oxidized in a tube furnace at 1100˚C to an oxide depth of approximately 200 nm.

*Bonding:* Before bonding, the etched and oxidized silicon wafer and a 4” 175-µm-thick Borofloat 33 wafer (Item #1837, University Wafer) were piranha cleaned in a 3:1 mixture of concentrated sulfuric acid to 30% hydrogen peroxide for 10 minutes at room temperature to ensure surface cleanliness and activation. Immediately before bonding, both wafers were activated using O_2_ plasma (150 W for Si and 100 W for SiO_2_) for 3 min in a March Jupiter III RIE tool (acquired by Nordson, Westlake, OH, USA). The silicon wafer was anodically bonded to the glass wafer using the Electronic Visions Wafer Bonding system. Both wafers were manually aligned on the chuck with three metal flags separating the two at the edges. The chuck was loaded into the tool chamber, which was closed and sealed. An automated program was initiated with the following steps: (1) The chamber was heated to 300˚C and pumped down to 10^-3^ mbar before removing the flags and compressing the wafers with a piston force of 300 N. (2) A fifteen-minute waiting period was programmed to allow for equilibration of the system and initial surface bonding. (3) A 500V potential was applied for 20 min. (4) The chamber was cooled to below 60˚C. Once cooled, the wafer was removed. This process was optimized to reduce wafer bow due to residual stress and resulted in a low number of defects (on average, less than one device per fifteen-device wafer).

*Well and Retaining Feature Etching:* To allow for etching at two depths, both the wells and retaining features needed to be patterned before etching either. To achieve this, the oxide layer on the wafer surface was used to create a buried etch mask and photoresist was used to create an initial etch mask. The same photoresist protocol as before was followed to coat the silicon side of the wafer with ~5 µm SPR220 photoresist and expose the second mask layer (retaining feature trenches) on the MLA 150 aligner. This was aligned to the channels using the previously etched alignment marks. It was developed and hard baked with the same settings. Before etching, the wafer was exposed to 100W O_2_ plasma for one minute to ensure wetting of small features. The wafer was submerged in Buffer HF Improved (Transene Electronic Chemicals, Danvers, MA, USA) for approximately two minutes until the oxide was etched completely through. The wafer was rinsed thoroughly and the photoresist was stripped using the same process as before. After degreasing and dehydration, a slightly modified recipe was used for the photoresist deep etch mask. The wafer was exposed to 150W O_2_ plasma for 2 minutes. A molecular vapor deposition tool (Applied Microstructures, Inc., Anchorage, AK, USA) was used to deposit a single layer of hexamethyldisilazane to promote photoresist adhesion. Photoresist was spun on at a reduced speed to yield an approximately 7 µm photoresist layer. It was soft baked for 2 minutes at 60˚C and 4 minutes at 110˚C before aligning and exposing the third mask layer (wells and ports) at 300 mJ/cm^2^ on the MLA 150. The exposure was completed in two equal doses to reduce bubble formation. After at least one hour, a post-exposure bake of 90 seconds at 110˚C was used before developing in a 1:5 water to AZ 400K solution. The wafer was hard baked at 80˚C for 10 minutes. With both masks complete, the wafer was DRIE etched in the STS Pegasus. As before, this included an O_2_ descum before the etch. To minimize the effect of notching as the oxide stop became exposed, the etch was completed in two parts. A high-speed Bosch process etch using the high-frequency (13.56 MHz) platen was run for about 27 minutes until the silicon was nearly completely etched through. Then, a second etch utilizing the low-frequency platen (380 kHz) platen was used to finish the etch. Complete etching was confirmed visually through a viewing window in the etch chamber. After the deep etch, the photoresist was stripped as in previous steps, leaving only the buried oxide mask. The wafer was degreased and dehydrated before the final etch recipe (the same smooth etch used for the channels) was used to etch approximately 5-µm-deep trenches to define the retaining features. Because this etch is relatively gentle and short, it did not significantly affect the previously etched features.

*Finishing:* After completing the main feature etching, the thermal oxide layer covering the channel was typically intact and some silicon grass remained near the edges of the well. A final RIE etch was completed using the Freon RIE (Oxford Instruments, Abingdon, UK). A CHF_3_ (24 sccm) and Ar (38 sccm) etch (90W, 30 mT) was run for 10 minutes to remove the remaining oxide layer. Finally, an Oxford PlasmPro 100 plasma-enhanced chemical vapor deposition tool (Oxford Instruments) was used to deposit a 200 nm oxide layer over the exposed silicon surface. The individual devices were separated along lines etched during the well and port etch.

**Table 1**

**Previous Hanging Drop Array Literature**

| Authors | Title (Year) | Feature size | Retention method | Connected drops? |
| --- | --- | --- | --- | --- |
| Potrykus et al.^1^ | Multiple-drop-array (MDA) technique for the large-scale testing of culture media variations in hanging microdrop cultures of single cell systems. I: The technique (1979) | ~4 mm* | None | No |
| Kim et al.^2^ | Multi-Well Chip for Forming a Uniform Embryoid Body in a Tiny Droplet with Mouse Embryonic Stem Cells (2007) | 1.6 mm | Hydrophobic interaction |  |
| Lee et al.^3^ | A Hollow Sphere Soft Lithography Approach for Long-Term Hanging Drop Methods (2010) | 1.4-5.5 mm | Hydrophobic interaction | No |
| Tung et al.^4^ | High-throughput 3D spheroid culture and drug testing using a 384 hanging drop array (2011) | 3 mm | Retention feature | No |
| Frey et al.^5^ | Reconfigurable microfluidic hanging drop network for multi-tissue interaction and analysis (2014) | 3.5 mm | Retention feature | Yes |
| Yazdi et al.^6^ | Adding the ‘heart’ to hanging drop networks for microphysiological multi-tissue experiments (2015) | 3.5 mm | Retention feature | Yes |
| de Groot et al.^7^ | Surface-tension driven open microfluidic platform for hanging droplet culture (2016) | 4 mm | Retention feature | Yes |
| Wu et al.^8^ | A PDMS-Based Microfluidic Hanging Drop Chip for Embryoid Body Formation (2016) | 1 mm | Hydrophobic interaction | Yes |
| Kuo et al.^9^ | Three-dimensional spheroid culture targeting versatile tissue bioassays using a PDMS-based hanging drop array (2017) | 1.4 mm | Hydrophobic interaction | No |
| Michael et al.^10^ | Surface-Engineered Paper Hanging Drop Chip for 3D Spheroid Culture and Analysis (2018) | 2-5 mm | Hydrophobic interaction | Yes |
| Aeby et al.^11^ | Microfluidics: Microfluidic Hydrogel Hanging-Drop Network for Long-Term Culturing of 3D Microtissues and Simultaneous High-Resolution Imaging (2018) | 2 mm | Retention feature | No |
| Zhao et al.^12^ | A 3D Printed Hanging Drop Dripper for Tumor Spheroids Analysis Without Recovery (2019) | 2.5 mm | Retention feature | No |
| Gao et al.^13^ | Fabrication of three-dimensional islet models by the geometry-controlled hanging-drop method (2019) | 3-7 mm | Retention feature | No |
| Shao et al.^14^ | Droplet Microarray on Patterned Butterfly Wing Surfaces for Cell Spheroid Culture (2019) | 0.1-3 mm | Hydrophobic interaction | No |
| Cho et al.^15^ | Development of a Novel Hanging Drop Platform for Engineering Controllable 3D Microenvironments (2020) | 4.6 mm | Retention feature | No |
| Huang et al.^16^ | A Dynamic Hanging-Drop System for Mesenchymal Stem Cell Culture (2020) | 5 mm | Retention feature | Yes |
| Park et al.^17^ | Microfluidic channel-integrated hanging drop array chip operated by pushbuttons for spheroid culture and analysis (2020) | ~3 mm* | Retention feature | Yes |
| Ganguli et al.^18^ | Three-dimensional microscale hanging drop arrays with geometric control for drug screening and live tissue imaging (2021) | 100-500 µm | Hydrophobic interaction | No |
| Fu et al.^19^ | Cutting and Bonding Parafilm® to Fast Prototyping Flexible Hanging Drop Chips for 3D Spheroid Cultures (2021) | 3-5 mm | Hydrophobic interaction | No |
| Sun et al.^20^ | A superhydrophobic chip integrated with an array of medium reservoirs for long-term hanging drop spheroid culture (2021) | 2 mm | Hydrophobic interaction | No |
| Liu et al.^21^ | A Novel SimpleDrop Chip for 3D Spheroid Formation and Anti-Cancer Drug Assay (2021) | ~1 mm* | Hydrophobic interaction | No |
| Su et al.^22^ | A Facile and Scalable Hydrogel Patterning Method for Microfluidic 3D Cell Culture and Spheroid-in-Gel Culture Array (2021) | 1.5-3 mm | Retention feature | No |
| Wu Jin et al.^23^ | A Microfluidic Hanging-Drop-Based Islet Perifusion System for Studying Glucose-Stimulated Insulin Secretion From Multiple Individual Pancreatic Islets (2021) | 2 mm | Retention feature | Yes |
| Boos et al.^24^ | Microfluidic Co-Culture Platform to Recapitulate the Maternal–Placental–Embryonic Axis (2021) | 3 mm | Retention feature | Yes |
| Jeong et al.^25^ | Flipped Well-Plate Hanging-Drop Technique for Growing Three-Dimensional Tumors (2022) | 6.86 mm* | Hydrophobic interaction | No |
| Rodoplu et al.^26^ | A microfluidic hanging drop-based spheroid co-culture platform for probing tumor angiogenesis (2022) | 2.5 mm^†^ | Retention feature | Yes |
| Struber et al.^27^ | Low-Cost Devices for Three-Dimensional Cell Aggregation, Real-Time Monitoring Microscopy, Microfluidic Immunostaining, and Deconvolution Analysis (2022) | 3 mm | Hydrophobic interaction | No |
| Kim et al.^28^ | All-in-one microfluidic design to integrate vascularized tumor spheroid into high-throughput platform (2022) | 0.5-2 mm | Hydrophobic interaction | No |
| Rousset et al.^29^ | Controlling bead and cell mobility in a recirculating hanging-drop network (2023) | 3.5 mm | Retention feature | Yes |
| Cui et al.^30^ | High‐throughput formation of miniaturized cocultures of 2D cell monolayers and 3D cell spheroids using droplet microarray (2023) | 1 mm | Hydrophobic interaction | No |
| Zhou et al.^31^ | Live Imaging of 3D Hanging Drop Arrays through Manipulation of Light-Responsive Pyroelectric Slippery Surface and Chip Adhesion (2023) | ~1.5 mm* | Hydrophobic interaction | No |
| Kim et al.^32^ | Reconfigurable Hanging Drop Microarray Platform for On-Demand Preparation and Analysis of Spheroid Array (2024) | 1 mm | Hydrophobic interaction | Yes^‡^ |

This table ignores subsequent publications with the same platform unless they substantially alter the fluidic design.

^*^not stated

^†^minor axis diameter

‡optional connection with reservoirs

**Table 1 References:**

1. Potrykus, I., Harms, C. T. & Lörz, H. Multiple-drop-array (MDA) technique for the large-scale testing of culture media variations in hanging microdrop cultures of single cell systems. I: The technique. *Plant Sci. Lett.* **14**, 231–235 (1979).

2. KIM, C. *et al.* Multi-Well Chip for Forming a Uniform Embryoid Body in a Tiny Droplet with Mouse Embryonic Stem Cells. *Biosci. Biotechnol. Biochem.* **71**, 2985–2991 (2007).

3. Lee, W. G., Ortmann, D., Hancock, M. J., Bae, H. & Khademhosseini, A. A Hollow Sphere Soft Lithography Approach for Long-Term Hanging Drop Methods. *Tissue Eng. Part C Methods* **16**, 249–259 (2010).

4. Tung, Y.-C. *et al.* High-throughput 3D spheroid culture and drug testing using a 384 hanging drop array. *Analyst* **136**, 473–478 (2011).

5. Frey, O., Misun, P. M., Fluri, D. A., Hengstler, J. G. & Hierlemann, A. Reconfigurable microfluidic hanging drop network for multi-tissue interaction and analysis. *Nat. Commun.* **5**, 4250 (2014).

6. Yazdi, S. R. *et al.* Adding the ‘heart’ to hanging drop networks for microphysiological multi-tissue experiments. *Lab. Chip* **15**, 4138–4147 (2015).

7. Groot, T. E. de, Veserat, K. S., Berthier, E., Beebe, D. J. & Theberge, A. B. Surface-tension driven open microfluidic platform for hanging droplet culture. *Lab. Chip* **16**, 334–344 (2016).

8. Wu, H.-W., Hsiao, Y.-H., Chen, C.-C., Yet, S.-F. & Hsu, C.-H. A PDMS-Based Microfluidic Hanging Drop Chip for Embryoid Body Formation. *Molecules* **21**, 882 (2016).

9. Kuo, C.-T. *et al.* Three-dimensional spheroid culture targeting versatile tissue bioassays using a PDMS-based hanging drop array. *Sci. Rep.* **7**, 4363 (2017).

10. Michael, Issac. J. *et al.* Surface-Engineered Paper Hanging Drop Chip for 3D Spheroid Culture and Analysis. *ACS Appl. Mater. Interfaces* **10**, 33839–33846 (2018).

11. Aeby, E. A., Misun, P. M., Hierlemann, A. & Frey, O. Microfluidics: Microfluidic Hydrogel Hanging-Drop Network for Long-Term Culturing of 3D Microtissues and Simultaneous High-Resolution Imaging (Adv. Biosys. 7/2018). *Adv. Biosyst.* **2**, 1870062 (2018).

12. Zhao, L. *et al.* A 3D Printed Hanging Drop Dripper for Tumor Spheroids Analysis Without Recovery. *Sci. Rep.* **9**, 19717 (2019).

13. Gao, B., Jing, C., Ng, K., Pingguan-Murphy, B. & Yang, Q. Fabrication of three-dimensional islet models by the geometry-controlled hanging-drop method. *Acta Mech. Sin.* **35**, 329–337 (2019).

14. Shao, C. *et al.* Droplet Microarray on Patterned Butterfly Wing Surfaces for Cell Spheroid Culture. *Langmuir* **35**, 3832–3839 (2019).

15. Cho, C.-Y. *et al.* Development of a Novel Hanging Drop Platform for Engineering Controllable 3D Microenvironments. *Front. Cell Dev. Biol.* **8**, (2020).

16. Huang, S.-W., Tzeng, S.-C., Chen, J.-K., Sun, J.-S. & Lin, F.-H. A Dynamic Hanging-Drop System for Mesenchymal Stem Cell Culture. *Int. J. Mol. Sci.* **21**, 4298 (2020).

17. Park, J., Kim, H. & Park, J.-K. Microfluidic channel-integrated hanging drop array chip operated by pushbuttons for spheroid culture and analysis. *Analyst* **145**, 6974–6980 (2020).

18. Ganguli, A. *et al.* Three-dimensional microscale hanging drop arrays with geometric control for drug screening and live tissue imaging. *Sci. Adv.* **7**, eabc1323 (2021).

19. Fu, J. J. *et al.* Cutting and Bonding Parafilm® to Fast Prototyping Flexible Hanging Drop Chips for 3D Spheroid Cultures. *Cell. Mol. Bioeng.* **14**, 187–199 (2021).

20. Sun, B., Zhao, Y., Wu, W., Zhao, Q. & Li, G. A superhydrophobic chip integrated with an array of medium reservoirs for long-term hanging drop spheroid culture. *Acta Biomater.* **135**, 234–242 (2021).

21. Liu, X. *et al.* A Novel SimpleDrop Chip for 3D Spheroid Formation and Anti-Cancer Drug Assay. *Micromachines* **12**, 681 (2021).

22. Su, C. *et al.* A Facile and Scalable Hydrogel Patterning Method for Microfluidic 3D Cell Culture and Spheroid-in-Gel Culture Array. *Biosensors* **11**, 509 (2021).

23. Wu Jin, P., Rousset, N., Hierlemann, A. & Misun, P. M. A Microfluidic Hanging-Drop-Based Islet Perifusion System for Studying Glucose-Stimulated Insulin Secretion From Multiple Individual Pancreatic Islets. *Front. Bioeng. Biotechnol.* **9**, (2021).

24. Boos, J. A. *et al.* Microfluidic Co-Culture Platform to Recapitulate the Maternal–Placental–Embryonic Axis. *Adv. Biol.* **5**, 2100609 (2021).

25. Jeong, Y., Tin, A. & Irudayaraj, J. Flipped Well-Plate Hanging-Drop Technique for Growing Three-Dimensional Tumors. *Front. Bioeng. Biotechnol.* **10**, (2022).

26. Rodoplu, D., Sierra Matahum, J. & Hsu, C.-H. A microfluidic hanging drop-based spheroid co-culture platform for probing tumor angiogenesis. *Lab. Chip* **22**, 1275–1285 (2022).

27. Struber, A. *et al.* Low-Cost Devices for Three-Dimensional Cell Aggregation, Real-Time Monitoring Microscopy, Microfluidic Immunostaining, and Deconvolution Analysis. *Bioengineering* **9**, 60 (2022).

28. Kim, Y. *et al.* All-in-one microfluidic design to integrate vascularized tumor spheroid into high-throughput platform. *Biotechnol. Bioeng.* **119**, 3678–3693 (2022).

29. Rousset, N. *et al.* Controlling bead and cell mobility in a recirculating hanging-drop network. *Lab. Chip* **23**, 4834–4847 (2023).

30. Cui, H. *et al.* High-throughput formation of miniaturized cocultures of 2D cell monolayers and 3D cell spheroids using droplet microarray. *Droplet* **2**, e39 (2023).

31. Zhou, S. *et al.* Live Imaging of 3D Hanging Drop Arrays through Manipulation of Light-Responsive Pyroelectric Slippery Surface and Chip Adhesion. *Nano Lett.* **23**, 10710–10718 (2023).

32. Kim, H. *et al.* Reconfigurable Hanging Drop Microarray Platform for On-Demand Preparation and Analysis of Spheroid Array. *Adv. Healthc. Mater.* **n/a**, 2400501.
